# Hypericin in the Light and in the Dark: Two Sides of the Same Coin

**DOI:** 10.3389/fpls.2016.00560

**Published:** 2016-05-06

**Authors:** Zuzana Jendželovská, Rastislav Jendželovský, Barbora Kuchárová, Peter Fedoročko

**Affiliations:** Department of Cellular Biology, Faculty of Science, Pavol Jozef Šafárik University in KošiceKošice, Slovakia

**Keywords:** hypericin, St. John's wort, anticancer activities, photodynamic therapy, photodynamic diagnosis, drug resistance

## Abstract

Hypericin (4,5,7,4′,5′,7′-hexahydroxy-2,2′-dimethylnaphtodianthrone) is a naturally occurring chromophore found in some species of the genus *Hypericum*, especially *Hypericum perforatum* L. (St. John's wort), and in some basidiomycetes (*Dermocybe* spp.) or endophytic fungi (*Thielavia subthermophila*). In recent decades, hypericin has been intensively studied for its broad pharmacological spectrum. Among its antidepressant and light-dependent antiviral actions, hypericin is a powerful natural photosensitizer that is applicable in the photodynamic therapy (PDT) of various oncological diseases. As the accumulation of hypericin is significantly higher in neoplastic tissue than in normal tissue, it can be used in photodynamic diagnosis (PDD) as an effective fluorescence marker for tumor detection and visualization. In addition, light-activated hypericin acts as a strong pro-oxidant agent with antineoplastic and antiangiogenic properties, since it effectively induces the apoptosis, necrosis or autophagy of cancer cells. Moreover, a strong affinity of hypericin for necrotic tissue was discovered. Thus, hypericin and its radiolabeled derivatives have been recently investigated as potential biomarkers for the non-invasive targeting of tissue necrosis in numerous disorders, including solid tumors. On the other hand, several light-independent actions of hypericin have also been described, even though its effects in the dark have not been studied as intensively as those of photoactivated hypericin. Various experimental studies have revealed no cytotoxicity of hypericin in the dark; however, it can serve as a potential antimetastatic and antiangiogenic agent. On the contrary, hypericin can induce the expression of some ABC transporters, which are often associated with the multidrug resistance (MDR) of cancer cells. Moreover, the hypericin-mediated attenuation of the cytotoxicity of some chemotherapeutics was revealed. Therefore, hypericin might represent another St. John's wort metabolite that is potentially responsible for negative herb–drug interactions. The main aim of this review is to summarize the benefits of photoactivated and non-activated hypericin, mainly in preclinical and clinical applications, and to uncover the “dark side” of this secondary metabolite, focusing on MDR mechanisms.

## Introduction

Hypericin (4,5,7,4′,5′,7′-hexahydroxy-2,2′-dimethylnaphtodiant hrone) is a naturally occurring compound synthesized by some species of the genus *Hypericum*. Hypericin was first isolated from *Hypericum perforatum* L. (Brockmann et al., 1939), commonly known as St. John's wort, which is one of the best characterized and most important representatives of this genus, because of its broad pharmacological activity (antidepressant, antimicrobial, anticancer, anti-inflammatory, wound healing, etc.) (reviewed in Kasper et al., 2010; Wölfle et al., 2014). Hypericin and its derivatives are accumulated in special morphological structures, so called dark nodules, occurring in the aerial parts of hypericin-producing *Hypericum* species. The newest data on interspecific variation in localization of hypericins and spatial chemo-profiling of hypericin in some *Hypericum* species were published recently (Kusari et al., 2015; Kucharikova et al., 2016).

In addition to St. John's wort, this secondary metabolite was found in several other *Hypericum* species (Kitanov, 2001; Ayan et al., 2004) and in some basidiomycetes (*Dermocybe* spp.) (Dewick, 2002; Garnica et al., 2003) or endophytic fungi growing in *Hypericum perforatum* (*Thielavia subthermophila*) (Kusari et al., 2008, 2009). As hypericin is a bioactive compound that is applicable in several medicinal approaches, its content has been evaluated in *in vitro* grown *Hypericum perforatum* and in its transgenic clones (Čellárová et al., 1997; Košuth et al., 2003; Koperdáková et al., 2009), or in *Hypericum* cultures exposed to various biotechnological applications that focused on their preservation or stimulation of secondary metabolite production (Urbanová et al., 2006; Bruňáková et al., 2015; reviewed in: Čellárová, 2011).

Hypericin is well-known as a potent natural photosensitizing agent with great potential in anticancer photodynamic therapy (PDT) and photodynamic diagnosis (PDD). Besides its antineoplastic action, light-dependent *in vitro* fungicidal (Rezusta et al., 2012; Paz-Cristobal et al., 2014) and bactericidal effects (Kashef et al., 2013; García et al., 2015) have also been reported. In addition, light-activated hypericin is considered to be an effective antiviral agent (Hudson et al., 1993; Prince et al., 2000). However, some clinical studies have revealed that high doses of hypericin can induce phototoxic skin reactions without showing any detectable antiviral or antiretroviral activity in patients with viral infections (Gulick et al., 1999; Jacobson et al., 2001). The controversy concerning the virucidal effect of hypericin was summarized in detail by Kubin et al. (2005).

However, the potential use of this secondary metabolite in medicine might be broader than currently thought. Although hypericin has been extensively studied mainly because of its photodynamic and photocytotoxic properties, it also possesses various positive or negative biological activities without being activated by light.

## Light-activated hypericin

Hypericin possesses several properties that make it a powerful fluorescent photosensitizer that is suitable for PDT and PDD—attractive applications for the treatment and detection of tumors. It possesses minimal or no toxicity in the dark (Thomas and Pardini, 1992; Vandenbogaerde et al., 1997; Miadokova et al., 2010; Jendželovská et al., 2014; Feruszová et al., 2016), accumulates preferentially in neoplastic tissues (Kamuhabwa et al., 2002; Noell et al., 2011) and generates reactive oxygen species (ROS) in the presence of light (at wavelengths around 600 nm) and oxygen (Diwu and Lown, 1993). Thus, hypericin represents a potent natural alternative to chemically synthesized photosensitizers.

### Hypericin in photodynamic therapy

PDT represents a non-invasive therapeutic approach that is beneficial in the treatment of various cancerous (reviewed in Agostinis et al., 2011) and even non-cancerous lesions and disorders (reviewed in Kim et al., 2015). In general, it is based on the combined action of a photosensitizer, light and molecular oxygen. PDT involves the administration of a non-toxic photosensitizer that preferentially accumulates in the target tissue, followed by its local illumination with harmless visible light of an appropriate wavelength, to activate and excite the photosensitizer. These photoreactions lead to the oxygen-dependent generation of cytotoxic ROS, resulting in cell death and tissue destruction. However, PDT is a multifactorial process and the degree of cellular photodamage depends on many factors, including cell permeability, the subcellular localization of the photosensitizer, the quantity of molecular oxygen, the light dose, the types of generated ROS and the attributes of cancer cells.

The exact mechanisms of cellular hypericin uptake are still unclear and require further investigation, but the results indicate that hypericin might be transported into or through cells via temperature-dependent diffusion (Thomas and Pardini, 1992; Sattler et al., 1997), partitioning, pinocytosis or endocytosis (Siboni et al., 2002). Concerning its subcellular redistribution, the co-labeling of cancer cells with hypericin and fluorescent dyes specific for cell organelles revealed that hypericin accumulates in the membranes of the endoplasmic reticulum, the Golgi apparatus, lysosomes and mitochondria (Agostinis et al., 2002; Ali and Olivo, 2002; Galanou et al., 2008; Mikeš et al., 2011). However, the cellular uptake and subcellular localization of hypericin might be affected by its lipophilicity, incubation concentrations and/or interaction with serum lipoproteins (Crnolatac et al., 2005; Galanou et al., 2008; Kascakova et al., 2008). In brief, upon light-activation, hypericin is efficient primarily in the generation of singlet oxygen (^1^O_2_; type II mechanism) and superoxide anion (${\text{O}}_{2}^{›-}$; type I mechanism) (Thomas et al., 1992; Diwu and Lown, 1993), which can ultimately lead to necrosis (Du et al., 2003b; Mikeš et al., 2007, 2009), apoptosis (Ali and Olivo, 2002; Mikeš et al., 2009), autophagy-associated cell death (Buytaert et al., 2006; Rubio et al., 2012) or even to immunogenic cell death (ICD) (Garg et al., 2012a). As type II ICD inducer (Garg et al., 2015a), HY-PDT represents a promising form of active immunotherapy (Galluzzi et al., 2014) owing to spatiotemporally defined emission of damage-associated molecular patterns (DAMPs) (Garg et al., 2012b, 2015b, 2016; Zheng et al., 2016).

The photocytotoxicity of hypericin is strongly oxygen-dependent, as no such effects are present in hypoxic conditions (Thomas and Pardini, 1992; Delaey et al., 2000). Nevertheless, the final response of hypericin-mediated PDT (HY-PDT) might also be affected by the ability of cells to overcome oxidative stress through the activity of various cytoprotective mechanisms, including cellular redox systems (Mikeš et al., 2011; Mikešová et al., 2013). Furthermore, the light-dependent inhibitory effect of hypericin against various enzymes engaged in the regulation of cell survival and proliferation (Ser/Thr kinases, tyrosine kinases, etc.) has been reported (reviewed in Kubin et al., 2005). These activities might also contribute to the cytotoxic and antiproliferative effects of HY-PDT. The exact mechanisms of action and the cellular aspects of HY-PDT have been outlined and summarized in several reviews (Agostinis et al., 2002; Theodossiou et al., 2009; Mikeš et al., 2013; Garg and Agostinis, 2014).

#### Preclinical and clinical assessment of HY-PDT efficacy and suitable conditions

Many *in vitro* studies have demonstrated the cytotoxicity of photoactivated hypericin in various cancer cell types (Xie et al., 2001; Head et al., 2006; Sacková et al., 2006; Mikeš et al., 2007; Koval et al., 2010; Mikešová et al., 2013; Kleemann et al., 2014). Moreover, recent *in vivo*, preclinical and clinical studies have indicated that HY-PDT might be an effective and relevant approach in the treatment of some skin tumors, carcinomas and sarcomas. In general, the depth of tumor destruction after PDT commonly ranges from a few mm to 1 cm, due to limited photosensitizer and light penetration through the tissues. Thus, PDT is effective mostly against superficial lesions and small tumors.

##### Clinical studies to test HY-PDT efficacy

To our knowledge, three clinical trials of HY-PDT applied to various skin tumors have been published to date (Table 1). In the first study, Alecu et al. (1998) tested the intralesional injection of hypericin with subsequent photoactivation with visible light in the treatment of basal cell carcinoma (BCC) (eleven patients) and squamous cell carcinoma (SCC) (eight patients) and found that HY-PDT was effective in the treatment of both skin disorders. A reduction in tumor size and the generation of a new epithelium at the surface of lesions following HY-PDT were observed. Moreover, as no necrosis or cell loss was evident in the surrounding healthy tissues and no side effects were observed, with the exception of mild erythema in five cases (two patients with SCC, three patients with BCC), HY-PDT-mediated tumor targeting was selective. The treatment resulted in a complete clinical response in one SCC patient and two BCC patients, but in the remaining patients, only a partial clinical response was observed. Thus, the efficacy of HY-PDT appeared to be dependent on the initial lesion size, the total dose of hypericin, or the frequency and duration of the therapy (Alecu et al., 1998). Several years later, the potential use of HY-PDT in the treatment of non-melanoma skin cancers was explored (Kacerovská et al., 2008). A complete clinical response was observed in 50% of patients with actinic keratosis (AK) 3 months after HY-PDT, and in 22% of patients with superficial BCC and 40% of patients with Bowen's disease (BD) 6 months after HY-PDT. However, in the case of AK, the percentage reduced to 29% 6 months after HY-PDT and only partial remission was observed in patients with nodular BCC. On the other hand, complete histological remission was evident in 80% of patients with BD (Kacerovská et al., 2008). Only the partial response rate and suboptimal success of HY-PDT could be caused by the limited penetration of the skin by hypericin and by its low concentration in the final extraction product. In the third clinical trial, Rook et al. (2010) tested HY-PDT as a potentially well-tolerated and effective therapeutic modality for the treatment of lymphocyte-mediated skin disorders: malignant mycosis fungoides (MF; the most common type of cutaneous T-cell lymphoma) and non-cancerous autoimmune psoriasis. The results were promising for both diseases. In the case of MF, HY-PDT led to an improvement in the treated lesions (a size reduction by at least 50%) in the majority of patients, whereas the placebo was ineffective. Moreover, hypericin was well tolerated by the patients, with only mild to moderate phototoxic skin reactions occurring after exposure to visible light. No serious adverse effects or events were observed (Rook et al., 2010). However, the authors themselves recommended a phase III study with a greater number of patients. All these clinical data indicate that topically applied hypericin, combined with its photoactivation, might be a promising and safe alternative for the treatment of some cancerous and non-cancerous skin disorders. However, as the effectiveness of HY-PDT depends on the hypericin concentration, its total dose, its rate of tissue penetration, the frequency and duration of the therapy, or on the grade of malignancy, more clinical trials are necessary to define the optimal conditions for the whole procedure.

**Table 1:** **Clinical studies to test HY-PDT efficacy**.

**Disease/No. of patients**	**Hypericin administration**	**Hypericin dosage**	**Light dose/Fluence rate**	**HY-PDT efficacy**	**References**
Squamous cell carcinoma/8	Intralesional injection	40–100 μg 3–5 times per week for 2–4 weeks;	86 J/cm^2^/24 mW/cm^2^	Reduction in tumor size, re-epithelization at the borders of the lesion, complete clinical remission in the case of one patient;	Alecu et al., 1998
Basal cell carcinoma/11		40–200 μg 3–5 times per week for 2–6 weeks		Reduction in tumor size, complete clinical remission in the case of two patients, no evident signs of tumor recurrence after 5 months	
Actinic keratosis/8 Basal cell carcinoma/21	On the lesion	Weekly for 6 weeks on average	75 J/cm^2^	50% complete clinical response (AK) 28% complete clinical response (superficial BCC) 11% complete histological response (superficial BCC) 67% partial clinical response (nodular BCC)	Kacerovská et al., 2008
Bowen's disease/5				40% complete clinical response (BD) 80% complete histological response (BD)	
Mycosis fungoides (T-cell lymphoma)/12	On the lesion	0.005–0.025 mg/cm^2^ twice-weekly for 6 weeks	8–20 J/cm^2^	58.3% of responsive patients (reduction in MF lesion size by 50% or more)	Rook et al., 2010
Psoriasis/11				54.6% of responsive patients	

AK, actinic keratosis; BCC, basal cell carcinoma; BD, Bowen's disease; MF, mycosis fungoides.

##### Preclinical *in vivo* studies to test HY-PDT effects and conditions

Many further studies to test HY-PDT efficacy have been performed using mouse or rat animal models (Table 2). Several *in vivo* studies indicate that HY-PDT might be a promising approach in the treatment of bladder carcinomas. Kamuhabwa et al. (2002) reported selective hypericin uptake in bladder tumors and subsequently, even HY-PDT-mediated tumor damage was observed without the destruction of normal tissue (Kamuhabwa et al., 2003). In both studies, female Fisher rats with an orthotopic superficial transitional cell carcinoma (TCC) were used as an experimental model and hypericin was administered directly into the bladder via the catheter. The instilled hypericin accumulated selectively in the bladder urothelial tumors and the normal urothelium (in a ratio of 12:1), but no hypericin was detected in normal bladder submucosa and muscle layers, which is an important factor to avoid underlying tissue damage. In addition, no hypericin was detected in plasma; thus, systemic side-effects should not appear (Kamuhabwa et al., 2002).

**Table 2:** **Preclinical *in vivo* studies to test HY-PDT effects and conditions**.

**Experimental model/Type of tumor (cell line)**	**Hypericin administration**	**Hypericin dose**	**Light dose/Fluence rate**	**HY-PDT effects**	**References**
Athymic nude mice/Epidermoid carcinoma (A431)	Intraperitoneal injection	2.5 mg/kg, 5 mg/kg	180 J/cm^2^	Tumor growth inhibition, reduced tumor mass	Vandenbogaerde et al., 1996
Athymic nude mice/Pancreatic carcinoma (MiaPaCa-2)	Intratumoral Injection	10 μg/mouse	2 doses of 200 J	Suppressed growth of subcutaneous and orthotopic tumors	Liu et al., 2000
DBA/2 mice/Lymphoma (P388)	Intraperitoneal injection	2, 5 or 20 mg/kg	120 J/cm^2^/100 mW/cm^2^	Reduced tumor mass and tumor size, prolonged survival time	Chen and de Witte, 2000
Nude mice/Prostate carcinoma (LNCaP)	Oral	5 mg/kg	30 mW	Tumor growth inhibition	Xie et al., 2001
C3H/Km mice/Fibrosarcoma (RIF-1)	Intravenous injection	5 mg/kg	120 J/cm^2^/100 mW/cm^2^	Tumor vasculature damage after 0.5 h DLI PDT resulting in complete tumor cure, apoptosis as a main form of cell death	Chen et al., 2001, 2002a,b
Fischer CDF (F344)/CrlBR rats/Bladder carcinoma (AY-27)	Intravenous injection	1 or 5 mg/kg	120 J/cm^2^/100 mW/cm^2^	Reduced tumor size, no measurable tumor mass 9–10 days after 0.5 h DLI PDT	Zupkó et al., 2001
C3H/DiSn mice/Fibrosarcoma (G5:1:13)	Intratumoral or intraperitoneal injection	5 mg/kg	180 J/cm^2^/150 mW/cm^2^	Reduced tumor volume, prolonged survival time, complete remission in smaller lesions (3 mm or less in size)	Čavarga et al., 2001
	Intraperitoneal injection	1 × 5 mg/kg, 2 × 2.5 mg/kg	168 J/cm^2^/70 mW/cm^2^	Higher efficiency of fractionated dose Vascular damage, formation of necrotic areas	Čavarga et al., 2005; Bobrov et al., 2007
Balb/c mice/Colon carcinoma (C26)	Intraperitoneal injection	5 mg/kg	60, 90 or 120 J/cm^2^/100 mW/cm^2^	Vascular damage, tumor necrosis (the depth of tumor necrosis increased with increased light dose)	Blank et al., 2002
Balb/c mice/Ehrlich ascites carcinoma	Intraperitoneal injection	40 mg/kg	50 mW/cm^2^	Prolonged survival time (75% of mice), no tumor recurrence (25% of survived mice)	Lukšienė and De Witte, 2002
Fischer rats/Bladder carcinoma (AY-27)	Instillation into the bladder	30 μM	6–48 J/cm^2^/25–50 mW/cm^2^	Selective urothelial tumor damage without destructive effects on detrusor musculature	Kamuhabwa et al., 2003
Balb/c nude mice/Nasopharyngeal carcinoma (HK-1)	Intravenous injection	2 mg/kg	120 J/cm^2^/226 mW/cm^2^	Inhibited tumor growth, tumor shrinkage, necrosis as a main form of cell death	Du et al., 2003a,b
		2 or 5 mg/kg	30 J/cm^2^/25 mW/cm^2^	Increased apoptosis and lower serum levels of VEGF after 6 h DLI PDT	Thong et al., 2006
Athymic nude mice/Squamous carcinoma (SNU1)	Intratumoral injection	10 μg per mg tumor	0–60 J/cm^2^	Regression of smaller tumors (under 400 mm^3^)	Head et al., 2006
Balb/c nude mice/Bladder carcinoma (MGH)	Intravenous injection	5 mg/kg	120 J/cm^2^/100 mW/cm^2^	Vascular damage after 0.5 h DLI PDT resulting in reduced tumor volume, increased expression of some angiogenic proteins after 6 h DLI PDT	Bhuvaneswari et al., 2008
Wistar-Furth rats/Pituitary adenoma (GH4C1)	Intraperitoneal injection	4 × 1 mg/kg	105–130 J/m^2^	Inhibited growth of smaller tumors (under 1 cm^3^), formation of apoptotic clusters	Cole et al., 2008
NMRI – HR-HR hairless mice/UV-induced small skin tumors	Topical application	0.1% in gelcream	40 J/cm^2^/20 mW/cm^2^	Full lesional necrosis resulting in total lesional clearance (44%), replacement of atypical AK cells by normal keratinocytes	Boiy et al., 2010
Balb/c mice/Colon carcinoma (CT26)	Intravenous injection	2.5 or 10 mg/kg	14 or 60 J/cm^2^/27 or 50 mW/cm^2^	Vascular damage after “low power PDT” resulting in complete tumor regression, prevention of new tumor growth after the re-challenge of cured mice with CT26 cells	Sanovic et al., 2011
NOD/LtSz-scid IL2Rγnull mice/Rhabdomyosarcoma (Rh30)	Intravenous injection	100 μg/mouse	—-	Induction of apoptosis in tumor cells	Urla et al., 2015
Balb/c mice/Colon carcinoma (CT26)	Subcutaneous injection of HY-PDT treated cells	150 nM	2.70 J/cm^2^	Tumor-rejecting anticancer vaccination effect after the re-challenge of cured mice with CT26 cells	Garg et al., 2012a, 2015b, 2016
Fischer 344 rats/Rat bladder carcinoma (AY27)	Subcutaneous injection of HY-PDT treated cells	150 nM	2.70 J/cm^2^	Absence of tumor-rejecting anticancer vaccination effect after the re-challenge of cured rats with AY27 cells	Garg et al., 2015b
C57BL/6 mice/Lewis lung carcinoma (LLC), Dendritic cells (DC) co-cultured with PDT-LLCs	Subcutaneous injection of HY-PDT treated cells	0.25 μM	1.85 J/cm^2^	Tumor-rejecting anticancer vaccination effect after the re-challenge of cured mice with LLC-Luc cells	Zheng et al., 2016

AK, actinic keratosis; DLI, drug-light interval; VEGF, vascular endothelial growth factor; –, the parameter was not provided by the authors.

Furthermore, photoactivated hypericin resulted in selective urothelial tumor damage, with tumor cells shrinking and detaching from the bladder wall, indicating that HY-PDT might be beneficial in the treatment of superficial carcinomas and premalignant changes in the bladder. The HY-PDT that was performed under suitable light conditions had no significant effects on the other bladder layers; nevertheless, 2–5% of tumor cells survived and were responsible for tumor regrowth (Kamuhabwa et al., 2003). However, following the results of *in vitro* study based on TCC-derived spheroids, the same authors suggested that hyperoxygenation could overcome this problem and might enhance the efficacy of HY-PDT (Huygens et al., 2005). In addition to the orthotopic tumor model, Liu et al. (2000) also used a xenograft model in their experiments. Human MiaPaCa-2 pancreatic adenocarcinoma cells were injected subcutaneously and orthotopically into the pancreatic bed of nude, athymic mice. To allow hypericin photoactivation in orthotopic pancreatic tumor nodules, mice underwent a laparotomy that was necessary for the positioning of the optical fiber. A significant decrease in growth of subcutaneous shoulder tumors (91.2% ± 2.3%) and even pancreatic tumor nodules (42.2% ± 8.1%) was observed 4 weeks after HY-PDT, indicating that intratumor hypericin and laser therapy might also be beneficial in the treatment of unresectable pancreatic cancer (Liu et al., 2000).

However, instead of more clinically relevant orthotopic tumor models, more *in vivo* studies have been performed to test the efficacy, conditions or responses of HY-PDT after the treatment, only in the murine or rat xenograft or allograft models of subcutaneous carcinomas or sarcomas. Various positive effects of HY-PDT involving the inhibition of tumor growth, a prolonged survival time of the treated animals, tumor necrosis, apoptosis or damage to the tumor vasculature were observed in mice bearing human epidermoid carcinoma (Vandenbogaerde et al., 1996), human prostate adenocarcinoma cells (Xie et al., 2001), human nasopharyngeal carcinoma cells (Du et al., 2003a,b; Thong et al., 2006), human squamous carcinoma cells (Head et al., 2006), human bladder carcinoma cells (Bhuvaneswari et al., 2008), human rhabdomyosarcoma cells (Urla et al., 2015), murine lymphoma cells (Chen and de Witte, 2000), murine colon adenocarcinoma cells (Blank et al., 2002; Sanovic et al., 2011), murine fibrosarcoma cells (Čavarga et al., 2001, 2005; Chen et al., 2001, 2002a,b; Bobrov et al., 2007) or murine Ehrlich ascites carcinoma cells (Lukšienė and De Witte, 2002) and in rats bearing rat bladder transitional bladder carcinoma (Zupkó et al., 2001) or rat pituitary adenoma cells (Cole et al., 2008) (Table 2). In addition, Blank et al. (2002) demonstrated the dependence of HY-PDT efficacy on the irradiation conditions (light dose and wavelength). Tumor necrosis was much more pronounced at 590 nm than at 550 nm and even increased when the light dose was raised from 60 to 120 J/cm^2^; however, the maximum depth of tumor necrosis was 9.9 ± 0.8 mm at 590 nm (Blank et al., 2002). Considering the relationship between HY-PDT efficacy and tumor volume, similar results were obtained in other studies. Head et al. (2006) and Cole et al. (2008) observed a regression or reduction in tumor size only in tumors smaller than 0.4 or 1 cm^3^, respectively, whereas larger tumors showed only a partial response followed by their regrowth (Head et al., 2006), or did not respond to the treatment (Cole et al., 2008). Thus, light penetration into the tissue appeared to be a limiting factor. However, Cole et al. (2008) also concluded that HY-PDT can be effective in the elimination of small solid tumor residues. Čavarga et al. (2001) also determined complete remission only in smaller tumors (3 mm or less in height), but the main aim of their study was to compare the impact of intraperitoneal and intratumoral hypericin injection on the effectiveness of HY-PDT. Both schedules of hypericin administration significantly reduced tumor volume and increased the survival rate of animals. However, considering the complete response, a higher HY-PDT efficacy was observed for hypericin that was administered intraperitoneally (44.4%) compared to intratumorally (33.3%) (Čavarga et al., 2001). Moreover, it was later demonstrated that a better therapeutic response was obtained after fractionated hypericin administration (two 2.5 mg/kg doses; 6 and 1 h before irradiation) than after a single hypericin dose (5 mg/kg; 1 or 6 h before irradiation) (Čavarga et al., 2005).

Furthermore, Chen et al. (2001) reported the correlation between hypericin biodistribution, HY-PDT efficacy and various administration–irradiation time intervals (drug–light intervals—DLIs). It was found that shortly (0.5 h) after the intravenous administration of hypericin (5 mg/kg), the photosensitizer was located preferentially within tumor blood vessels. At 6 h after injection, maximum intratumoral hypericin content was evident and only poor fluorescence was detected in the tumor vasculature. Despite high tumor hypericin levels, no tumor cure was observed after longer DLI HY-PDT (6 h) treatment. However, the efficacy of PDT was maximal (100% tumor cure) when irradiation was performed at 0.5 h after hypericin administration (short DLI), indicating strong HY-PDT-induced damage to the tumor vasculature (Chen et al., 2001). Similar results were obtained by Zupkó et al. (2001). In TCC tumors, the outcome of HY-PDT was highly dependent on DLI (0.5, 6, or 24 h). The strongest effect, which resulted in no tumor regrowth in some rats, was evident after 0.5 h DLI PDT. At the same time, the highest hypericin concentration was detected in the plasma, indicating that PDT-mediated tumor vascular damage was responsible for its antineoplastic action (Zupkó et al., 2001). The antivascular and strong antitumoral effects of short DLI HY-PDT were also subsequently confirmed in the murine fibrosarcoma model (Chen et al., 2002a,b). Damage to tumor vessels was also detected following long DLI HY-PDT treatments, but many viable tumor cells were present, especially at the tumor periphery, indicating that this PDT modality only induced partial vascular collapse (Chen et al., 2002a). In agreement with these results, Bobrov et al. (2007) also observed primary vascular damage after HY-PDT with either a single dose (5 mg/kg; 1 or 6 h before irradiation) or fractionated hypericin administration (two 2.5 mg/kg doses; 6 and 1 h before irradiation), which subsequently progressed to tumor tissue necrosis. The preference of HY-PDT-mediated vasculature or cellular damage appears to be dependent on the distribution and accumulation of the photosensitizer; thus, greater vascular destruction is expected after short DLI PDT and more direct killing of tumor cells is expected after long DLI PDT. However, because the damage to tumor vessels can have devastating consequences for whole tumor mass, the targeting of the tumor vasculature via short DLI PDT might be more effective in the treatment of solid tumors than a long DLI PDT. Moreover, apoptosis was the main form of cell death responsible for tumor eradication following short DLI HY-PDT (Chen et al., 2002b). In contrast, Thong et al. (2006) observed significantly more apoptosis after long DLI PDT (6 h) compared to short DLI PDT (1 h). However, different irradiation conditions and tumor models were used in both studies: whereas Chen et al. (2002b) photoactivated hypericin with a light dose of 120 J/cm^2^ delivered at a fluence rate of 100 mW/cm^2^, a lower fluence rate HY-PDT (light dose of 30 J/cm^2^, fluence rate of 25 mW/cm^2^) was applied by Thong et al. (2006). The results indicate that long DLI PDT might also be effective in the induction of programmed cell death, but only under low fluence rate conditions. Moreover, lower serum levels of vascular endothelial growth factor (VEGF) were detected after HY-PDT using a long DLI and a low fluence rate, which can reduce the risk of new tumor vasculature formation (Thong et al., 2006).

As PDT-mediated tissue damage can lead to various cellular and molecular responses, Bhuvaneswari et al. (2008) examined the potential anti-angiogenic vs. angiogenic properties of short (0.5 h) and long (6 h) DLI HY-PDT. Both HY-PDT scenarios led to a reduction in tumor volume, but the effect was much more pronounced for a short DLI. These findings agree with the above-mentioned results (Chen et al., 2001, 2002a,b) and suggest the destruction of the tumor vasculature after short DLI HY-PDT. However, in addition to its antitumor activities, cellular-targeted long DLI HY-PDT induced the expression of some angiogenic proteins in tumor tissue, including VEGF, tumor necrosis growth factor-α (TNF-α) and interferon-α (INF-α), which potentially lead to the formation of new vessels (Bhuvaneswari et al., 2008). In subsequent studies, the efficacy of HY-PDT was enhanced using monoclonal antibodies against VEGF and the epidermal growth factor receptor (EGFR) (Bhuvaneswari et al., 2010, 2011). For short DLI HY-PDT, similar results were obtained by Sanovic et al. (2011) in mice bearing CT26 colon carcinoma cells. Complete tumor regression was observed after “low-power HY-PDT” (a hypericin dose of 2.5 mg/kg, a short DLI of 0.5 h, a light dose of 14 J/cm^2^ and a fluence rate of 27 mW/cm^2^), as no visible or palpable tumors were detected for at least 60 days after the treatment. In contrast, all mice exposed to “high-power HY-PDT” (a hypericin dose of 10 mg/kg, a short DLI of 1 h, a light dose of 60 J/cm^2^ and a fluence rate of 50 mW/cm^2^) died 2 days after the treatment as a consequence of internal bleeding. Thus, both HY-PDT modalities with short DLIs appeared to preferentially target the vessels, but a higher hypericin concentration and light dose produced a much stronger response. These results suggest that “low-power HY-PDT” is strong enough to completely eliminate tumors by damaging their vasculature. Moreover, the re-challenge of cured mice with tumorigenic CT26 cells did not result in new tumor growth, indicating the HY-PDT-mediated induction of the antitumor immune response (Sanovic et al., 2011). HY-PDT-mediated induction of anticancer immunity was also observed in the studies utilizing different *in vivo* experimental models. Immunization of BALB/c mice with “dying or dead” colon carcinoma CT26 cells prevented the tumor growth at the rechallenge site treated with live CT26 tumor cells. Approximately 70–85% of the mice immunized with HY-PDT treated CT26 cells efficiently rejected the formation of CT26-derived tumors at challenge site (Garg et al., 2012a, 2015b, 2016). Activation of adaptive immune system was also detected in immunocompetent C57BL/6 mice immunized with HY-PDT treated Lewis lung carcinoma (LLC) cells and LLC cells co-cultured with dendritic cells (Zheng et al., 2016). The results of these three independent experimental groups suggest great potential of HY-PDT in development of anticancer vaccines.

Similar results as in the case of partial remission in patients with nodular BCC (Kacerovská et al., 2008) were obtained in an *in vivo* study using hairless mice with UV-induced non-melanoma skin tumors (AK, SCC) as an experimental model (Boiy et al., 2010). Photoactivated hypericin induced a total and partial response in 44 and 22% of lesions (diameter of 1–2 mm), respectively, with evident lesional necrosis and the replacement of atypical AK cells for normal keratinocytes. However, 33% of lesions were non-responsive to HY-PDT. Tumor penetration and the selectivity of topically applied hypericin was also limited by mouse skin, as the accumulation of hypericin was highest in the outermost epidermal layer and lower hypericin levels were detected in the rest of the epidermis and the dermis (Boiy et al., 2010).

Some preclinical and clinical results gave relatively disappointing results and in some cases, the efficacy of HY-PDT was not as high as expected. The above-mentioned studies contributed to a better understanding of HY-PDT-induced responses and highlighted the importance of optimizing the conditions, such as suitable hypericin administration, light dose, fluence rate, or time intervals and implicated HY-PDT as a promising anticancer therapeutic approach; however, more clinically relevant studies and trials are required for the implementation of HY-PDT into clinical practice.

### Hypericin in photodynamic diagnosis

In addition to their therapeutic abilities, most photosensitizers are also potent diagnostic agents. The main principle and relevance of PDD is to enhance the contrast between neoplastic and surrounding healthy tissue, which should contribute to the surgical clearance of the whole tumor mass or its small residues. Due to the fluorescent properties of hypericin and its specificity for neoplastic tissue, hypericin-mediated PDD (HY-PDD) is being tested for various clinical uses, including optical tumor imaging, and the targeting, monitoring or detection of tumor stages and grades. To date, fluorescence diagnosis using hypericin has been clinically tested in bladder, head and neck cancers or gliomas (Table 3).

**Table 3:** **Clinical studies to test HY-PDD efficacy, sensitivity and specificity**.

**No. of patients**	**Hypericin administration**	**Hypericin dose**	**Fluorescence excitation**	**HY-PDD efficacy**	**References**
**BLADDER CANCER**					
40	Instillation into the bladder	8 μM (40 ml)	FE/blue light	93% sensitivity and 98.5% specificity (for CIS)	D'Hallewin et al., 2000
87	Instillation into the bladder	8 μM (40 ml)	FE/blue light	94% sensitivity and 95% specificity (for CIS)	D'Hallewin et al., 2002
30	Instillation into the bladder	8 μM (50 ml)	FE/blue light	Fluorescence intensity increased with the stage and grade of cancer (normal < inflammation < grade 1 TCC < grade 2 TCC < CIS < grade 3 TCC)	Olivo et al., 2003b
41	Instillation into the bladder	8 μM (40 ml)	FE/violet light	Higher sensitivity (82%) compared to conventional WLE (62%)	Sim et al., 2005
57	Instillation into the bladder	0.25 mg HY + 25 mg PVP (50 ml)	FE/blue light	Higher overall sensitivity (95%) compared to conventional WLE (85%), fewer overlooked malignant lesions compared to WLE, sensitivity increased with the grade of cancer	Kubin et al., 2008
40	Instillation into the bladder	75 or 225 μg PVP-hypericin (50 ml)	FE/blue light	very strong fluorescence of 225 μg PVP-hypericin (120 and 60 min), optimal fluorescence of 225 μg PVP-hypericin instilled for 30 min, insufficient fluorescence of 75 μg and 225 μg PVP-hypericin (15 min)	Straub et al., 2015
8	Instillation into the bladder	8 μM (40 ml)	FM/380–425 nm	*Ex vivo* urine fluorescence cytology—fluorescence detected in all eight tumor cases	Pytel and Schmeller, 2002
29 urine samples	*Ex vivo* staining of sediment extracted from voided urine	Concentration not given (1 ml)	CFM/488 nm Argon laser	Higher fluorescence intensity in tumor cells than in cells from normal urine and in high-grade tumors than in low-grade tumors	Olivo et al., 2003a
21	*Ex vivo* staining of sediment extracted from voided urine	Concentration not given (1 ml)	CFM/488 nm Argon laser	Higher fluorescence intensity in tumor cells than in cells from normal urine	Fu et al., 2007
**GLIOMA**					
5	Intravenous injection	0.1 mg/kg	NM/blue light	Tumor fluorescence clearly distinguishable from normal brain tissue, high specificity (100 and 90%) and sensitivity (91 and 94%)	Ritz et al., 2012
**HEAD AND NECK CANCER**					
23	Oral rinsing	8 μM (100 ml)	FE/blue light	Distinguishing between various types of oral cancer (red-to-blue ratio), 90% and higher specificity and sensitivity (red-to-blue ratio)	Thong et al., 2009
2	Oral rinsing	8 μM (100 ml)	CLE/488 nm Argon laser	3-D visualization of human buccal mucosa at the surface and approximately 15 μm below the surface	Thong et al., 2012
27	*Ex vivo* tissue staining	8 μM	pCLE/568 nm laser diode	longer time interval for sufficient *ex vivo* staining (at least 30 min), the anomalies of keratinization not stained	Abbaci et al., 2015

CIS, carcinoma in situ; CFM, confocal fluorescence microscopy; CLE, confocal laser endomicroscopy; FE, fluorescence endoscopy; FM, fluorescence microscopy; HY, hypericin; NM, neurosurgical microscopy; pCLE, probe-based CLE; PVP, polyvinylpyrrolidone; TCC, transitional cell carcinoma; WLE, white-light endoscopy.

#### HY-PDD in the detection and identification of bladder cancer

In most published clinical studies, HY-PDD was applied to bladder tumors. As already mentioned in the HY-PDT section, Kamuhabwa et al. (2002) (Table 4) revealed selective hypericin uptake in rat bladder tumors using *in situ* laser-induced fluorescence (LIF) and fluorescence microscopy. The results suggest that hypericin is very beneficial in visualization and distinguishing the tumor mass from normal tissue using various fluorescent techniques. Thus, hypericin could be used not only in PDT, but also in the PDD of superficial bladder tumors.

**Table 4:** **Preliminary data for clinical HY-PDD applications and *in vivo* studies concerning hypericin accumulation**.

**Experimental model/Type of tumor (cell line)**	**Hypericin administration**	**Hypericin dose**	**Fluorescence excitation**	**Hypericin accumulation and fluorescence**	**References**
Fischer rats/Bladder carcinoma (AY-27)	Instillation into the bladder	8 or 30 μM	LIF/410 nm Krypton laser FM/525/50 nm	Intense fluorescence in tumor tissue and faint fluorescence in normal bladder tissue (ratio 12:1), no fluorescence in submucosa and muscle layers	Kamuhabwa et al., 2002
		30 μM PVP-hypericin	FM/510–560 nm	Higher accumulation (3.5-fold more) in malignant tissue than in normal urothelium	Vandepitte et al., 2010
Wistar rats/Glioma (C6)	Intravenous injection	5 mg/kg	FM/510–550 nm	Higher accumulation in glioma than in normal brain tissue and infiltration zone	Noell et al., 2011
VMDk mice/Glioma (SMA-560)	Intravenous injection	2.5 mg/kg	FM/510–550 nm FME/405 nm	Time-dependent accumulation in glioma cells (maximal uptake—6 h after administration), FME—fluorescence detection also in intracerebral and extracranial gliomas and in brain vessels	Noell et al., 2013
NOD/LtSz-scid IL2Rγnull mice/Rhabdomyosarcoma (Rh30)	Intravenous injection	100 μg/mouse	FL/blue light	Tumor fluorescence clearly distinguishable from normal healthy tissue	Urla et al., 2015

CLE, confocal laser endomicroscopy; FL, fluorescence laparoscopy; FM, fluorescence microscopy; FME, fluorescence microendoscopy; LIF, laser-induced fluorescence technique.

At about the same time, the first clinical studies were performed by D'Hallewin et al. (2000, 2002), who examined the fluorescence-based detection of flat bladder carcinomas *in situ* (CIS) and in papillary non-invasive bladder tumors after the intravesical instillation of hypericin (at least 2 h). The fluorescence emission was induced using fluorescence endoscopy (FE) under blue-light illumination. Hypericin accumulated selectively in tumor cells and papillary and flat lesions showed red fluorescence, whereas no fluorescence was evident in the normal bladder tissue. Subsequently, biopsies were taken from fluorescent regions for microscopic analyses. The results of both clinical studies suggested that HY-PDD has a high sensitivity and specificity for the detection of bladder cancer (D'Hallewin et al., 2000, 2002). Moreover, the results obtained by confocal microscopy, white-light endoscopy (WLE) and histopathology revealed that the intensity of hypericin fluorescence increased with the stage and grade of bladder cancer (normal bladder tissue < inflammation in the bladder < grade 1 TCC < grade 2 TCC < CIS < grade 3 TCC) (Olivo et al., 2003b). Therefore, HY-PDD could be used as a diagnostic aid to the histopathology of bladder tumors.

Nowadays, bladder lesions are conventionally diagnosed under WLE followed by biopsies that are necessary for the histological examination of suspicious tissues. However, in previous studies, some lesions were barely visible or were absent in white light (D'Hallewin et al., 2000, 2002), thus, WLE appears not to be sensitive enough to reveal all CIS lesions and leads to a high risk of missing the tumor. Therefore, another clinical study compared the WLE and HY-PDD methods (Sim et al., 2005). Hypericin was instilled into the bladder (for 2 h) and immediately after WLE, FE (violet light) was used to induce fluorescence emission in the same bladder regions. Despite the comparable specificity of both approaches (91% for HY-PDD, 98% for WLE), HY-PDD was more sensitive (82%) than WLE (62%) (Sim et al., 2005), suggesting that hypericin might be very potent in the labeling and early detection of flat superficial bladder tumors. Similar results were obtained by Kubin et al. (2008) using polyvinylpyrrolidone (PVP) bound to hypericin as a new water-soluble formula for the improvement of hypericin-mediated bladder cancer detection and diagnosis. Hypericin-PVP was intravesically instilled 1–2 h prior to FE. The overall sensitivity of PDD with PVP-hypericin (95%) was significantly higher than WLE (85%). The maximum contrast in sensitivity was evident in the case of CIS (100% for PDD vs. 33% for WLE) and dysplasia (85% for PDD vs. 31% for WLE) (Kubin et al., 2008).

As the PVP–hypericin complex represents a potent water-soluble PDD agent without the necessity of binding to serum proteins, its biodistribution (Vandepitte et al., 2010) and optimal dosage and instillation time were evaluated (Straub et al., 2015) in tumor-bearing rats and in patients with bladder cancer, respectively. Vandepitte et al. (2010) (Table 4) demonstrated the uniform distribution of instilled PVP–hypericin in all cell layers of the malignant urothelium, whereas its penetration into the normal bladder epithelium was very limited. Straub et al. (2015) tested various combinations of PVP–hypericin dosage (75 and 225 μg) and instillation time (15, 30, 60, and 120 min) to identify the optimal PDD conditions. Even though the fluorescence of 225 μg PVP-hypericin instilled for 120 and 60 min was very strong, the shorter instillation time (30 min) for 225 μg PVP–hypericin was evaluated as optimal. A lower photosensitizer dose (75 μg) and 15 min with a dose of 225 μg were insufficient to detect the lesions (Straub et al., 2015). The authors established the most suitable dosage and instillation time of PDD with PVP–hypericin, but they suggest a larger phase IIB study should be performed to determine the sensitivity and specificity of these optimal conditions.

All the above-mentioned results indicate that HY-PDD is highly sensitive in the detection of early bladder cancer and could be routinely used as a diagnostic approach. Moreover, no photobleaching during FE and resection or side effects were detected (D'Hallewin et al., 2002; Olivo et al., 2003b; Sim et al., 2005; Kubin et al., 2008). Kamuhabwa et al. (2005) also conclude that either photosensitization or systemic side-effects should not be expected in patients after intravesical hypericin administration, as the hypericin concentration in plasma was below the detection limit (< 6 nM).

Another common method used for bladder cancer diagnosis is *ex vivo* urine cytology, which microscopically analyzes the exfoliated bladder cells from voided urine. This diagnostic technique is non-invasive and less time-consuming than taking biopsy specimens. However, its sensitivity to detect early-stage or low-grade cancer is relatively low. Thus, several teams have focused on a technique that combines HY-PDD and urine cytology (Pytel and Schmeller, 2002; Olivo et al., 2003a; Fu et al., 2007). In the first study conducted by Pytel and Schmeller (2002), voided urine was analyzed in eight patients following intravesically instilled hypericin (for at least 1 h). Even though the number of patients was quite low, hypericin fluorescence was detected in all cases of bladder cancer. On the contrary, Olivo et al. (2003a) and Fu et al. (2007) performed HY-PDD-mediated urine cytology without intravesical instillation of hypericin. In both studies, sediments extracted from patient urine samples were incubated with hypericin in the dark for 15 min and were subsequently analyzed using confocal fluorescence microscopy. The overall fluorescence intensity of the urothelial cells was significantly higher in urine from early-grade TCC than in normal samples, which enabled the differentiation between normal and early bladder cancer specimens (Olivo et al., 2003a). This finding was later confirmed through a diagnostic algorithm (Fu et al., 2007). Moreover, fluorescence was even higher in high-grade tumors than in low-grade tumors (Olivo et al., 2003a). The results indicate that *ex vivo* fluorescence cytology using hypericin might be a promising diagnostic method for the detection and identification of early and low-grade bladder cancer.

#### HY-PDD in the detection of gliomas

It is well-known that malignant gliomas are tumors with a very poor prognosis and their complete resection significantly improves and extends the survival of patients. Thus, in these cases, the enhancement of the contrast between tumor and surrounding healthy tissue would be very beneficial for surgeons.

In the first *in vivo* study, Noell et al. (2011) (Table 4) investigated the accumulation of hypericin in tumors arising from intracerebrally implanted C6 glioma cells, in the zones surrounding the tumors and in healthy brain tissue. Hypericin was injected intravenously and its uptake was maximal 24 h after injection. Considering tissue autofluorescence, the ratios of fluorescence intensities were as follows: tumor:infiltration zone:normal tissue = 19.8:2.5:1.0. Because hypericin accumulation was significantly higher in the tumor than in normal tissue, it could be effectively used as a fluorescence marker for glioma detection (Noell et al., 2011). According to these promising preliminary results, the hypericin-mediated visualization of tumor tissue during its surgical resection was examined in five patients with recurrent glioblastomas (Ritz et al., 2012). Hypericin was injected intravenously 6 h prior to the surgical procedure, which was performed using a neurosurgical microscope under switchable white- and blue-light modes. Malignant tumor tissue (red fluorescence) was clearly distinguishable from the healthy brain tissue (blue color) in all patients and the margins of the tumors showed weaker pink fluorescence. Moreover, specimens were taken for histological evaluation, which was carried out by two neuropathologists and showed 100 and 90% specificity and 91 and 94% sensitivity. The obtained results suggest that HY-PDD is well-tolerated and represents a method that is sufficiently sensitive and specific for the intraoperative visualization of malignant gliomas (Ritz et al., 2012).

Furthermore, time-dependent hypericin uptake was investigated and observed in a subcutaneous glioma mouse model using microendoscopy, an approach that is not designed for microsurgical tumor resection, but is very useful for applications such as the visualization of different tissue compartments, the identification of vessels or the detection of optimal regions for biopsy. To verify the potential to detect intracerebral gliomas using microendoscopy, tumor cells were also implanted into the brain. After craniotomy, hypericin fluorescence was detected in intracerebral and extracranial gliomas and also in the vessels located in the cortical surface of the contralateral hemisphere (Noell et al., 2013) (Table 4).

#### HY-PDD in the detection and visualization of other types of malignancies

In addition to the above-mentioned studies, HY-PDD was also examined in mice bearing rhabdomyosarcoma (Urla et al., 2015) and in patients with various types of head and neck cancer (Thong et al., 2009, 2012; Abbaci et al., 2015) (Tables 3, 4).

Similarly to bladder cancer, lesions in the oral cavity are conventionally diagnosed using WLE and histopathology. However, the results obtained by Thong et al. (2009) demonstrate the great potential of hypericin for the diagnosis of various oral cancer types (hyperplasia, cellular pleomorphic adenoma of the palate, dysplasia, SCC). After oral rinsing with hypericin solution (over 30 min), FE was performed and the captured images were analyzed using several parameters. Firstly, the selective uptake of hypericin in tumor tissue was confirmed. Moreover, an increase in the red-to-blue fluorescence intensity ratio was evaluated from normal tissue (0.3) to hyperplasia (1.0) to SCC (2.0), which makes this parameter suitable for distinguishing between these tissue types with high specificity and sensitivity (over 90%) (Thong et al., 2009). Subsequently, the endomicroscopy imaging technique was improved by the same research team (Thong et al., 2012). Preliminary study already suggested that confocal laser endomicroscopy (CLE) might be a potent approach for the surface and subsurface imaging of oral cavity tissues using various fluorescent dyes in both animal and human models (Thong et al., 2007). Recently, a computing system was interfaced to CLE, which enables the 3-D fluorescence visualization of the oral cavity in real-time (Thong et al., 2012). This system could be integrated into current techniques for oral cancer diagnosis and might ultimately lead to better clinical outcomes.

The fluorescent properties of hypericin and four additional fluorescent dyes were also tested for their ability to characterize normal and cancerous head and neck tissue; however, the staining procedure was performed *ex vivo* on fresh samples obtained from head and neck surgeries (glossectomy, pharyngolaryngectomy, laryngectomy, etc.). Hypericin accumulated in the cytoplasm of normal and tumor cells, but was the only fluorescent dye that did not stain the anomalies of keratinization. Thus, the authors conclude that hypericin might not be a suitable photosensitizer for use in such head and neck specimens (Abbaci et al., 2015).

However, more promising *in vivo* results were obtained for intra-operative HY-PDD of rhabdomyosarcoma. In the preclinical study conducted by Urla et al. (2015), mice were injected intraperitoneally with human alveolar rhabdomyosarcoma cells and 3 weeks later, hypericin was administered intravenously. After 24 h, conventional and fluorescence laparoscopy were performed and the tumors were surgically resected using hypericin-mediated red fluorescence as guidance. Tumor specimens were processed for histological analyses. Conventional laparoscopy revealed 24 tumors (ranging in size from 1.6 to 13.5 mm) and 28 tumors were detected only by fluorescence laparoscopy (0.5–11 mm). The results indicate that intraoperative HY-PDD is more sensitive than conventional laparoscopy and can clearly distinguish rhabdomyosarcoma from healthy tissue (Urla et al., 2015). Moreover, the authors inform about clinical trial that will be initiated in children with advanced-stage rhabdomyosarcoma.

## Hypericin in dark conditions or the effects of hypericin without light-activation

Although hypericin has been extensively studied mainly because of its photodynamic and photocytotoxic properties, it also possesses various positive and negative biological activities without light-activation.

As some *in vitro* studies revealed cytotoxic or growth-inhibitory effects of non-activated hypericin (Blank et al., 2001, 2003; Berlanda et al., 2010; Besic Gyenge et al., 2012) and antiproliferative (Blank et al., 2001) and antimetastatic (Blank et al., 2004) activities *in vivo* and antiangiogenic actions *in vitro* (Martínez-Poveda et al., 2005) have been described, hypericin might also show antitumor potential in the absence of light. Moreover, one clinical study has demonstrated antiglioma activity of hypericin in dark conditions (Couldwell et al., 2011). However, the potential use of this natural compound in medicine might be broader. Regarding its avidity to necrotic tissues, the radiolabeled hypericin derivative ([^123^I]iodohypericin) can be used for radio-imaging of numerous necrosis-related pathologies (acute myocardial infarction, liver infarction) (Ni et al., 2006; Fonge et al., 2008), including solid tumors (Van de Putte et al., 2012).

On the other hand, hypericin can be associated with a decrease in the chemosensitivity of cancer cells because of its ability to induce the expression of some ATP-binding cassette (ABC) transporters, which are well-known multidrug resistance (MDR) components (Jendželovský et al., 2009; Jendželovská et al., 2014; Kuchárová et al., 2015). In addition, the hypericin-mediated attenuation of the cytotoxicity of some chemotherapeutic agents was demonstrated (Jendželovská et al., 2014).

### Non-activated hypericin and its potential antitumor activity

Hypericin-mediated photocytotoxic effects have always been a relevant and attractive issue for researchers in the field of oncology; however, the abilities of hypericin in the absence of light-activation have not been studied as intensively. Although several studies indicate that hypericin might possess some anticancer activities even in dark conditions (Table 5).

**Table 5:** **Anticancer effects of hypericin in dark conditions**.

**Experimental model**	**Hypericin doses and administration**	**Effects of non-activated hypericin**	**References**
***IN VITRO*** **STUDIES**			
Murine breast adenocarcinoma cell line (DA3) Murine anaplastic SCC cell line (SQ2)	0.065–10 μM (24 h), 0.2–20 μM (72 h), 0.6 and 6 μM	Mild decrease in cell viability detected by MTT assay (24 h, SQ2 cells), Significant decrease in cell viability detected by Hemacolor assay (72 h), Decrease in DNA synthesis detected by ^3^H-thymidine incorporation (72 h)	Blank et al., 2001
Murine melanoma cell line(B16.F10)	1–40 μM	Cytostasis detected by BrdU incorporation assay (72 h; doses ≤ 10 μM), Reduced cell viability detected by Hemacolor assay (72 h; doses > 10 μM); G2/M cell cycle arrest, formation of enlarged polynucleated cells and no evidence of apoptosis indicating mitotic cell death; enhanced ubiquitinylation of Hsp90 resulting in increased destabilization of its client proteins (p53, Cdk4, Plk, Raf-1)	Blank et al., 2003
Bovine aorta endothelial cells (BAE)	Range of concentrations, 5, 10 or 20 μM	Inhibition of some key steps of angiogenesis (decrease in urokinase extracellular level; inhibition of: endothelial cells proliferation, endothelial tube formation, migration and invasive capability of endothelial cells)	Martínez-Poveda et al., 2005
Human epidermoid carcinoma cell line (A431)	Range of concentrations (up to 100 μM)	Antiproliferative and/or cytotoxic effect detected by MTT assay (concentrations higher than 3.13 μM)	Berlanda et al., 2010
Human head and neck SCC carcinoma cell lines (UMB-SCC 745, UMB-SCC 969)	0.6–10 μg/ml	Antiproliferative effect detected by BrdU cell proliferation assay (all applied concentrations), no influence on RNA integrity, initial DNA damage (recovered after 3 h)	Besic Gyenge et al., 2012
***IN VIVO*** **STUDIES**			
Balb/c mice/Murine breast carcinoma (DA3) Murine SCC (SQ2)	200, 100 and 50 μM; intraperitoneal injection	Reduced volume of DA3-derived tumors (66% at 20 days after beginning of treatment), prolonged survival time (both DA3 and SQ2 models)	Blank et al., 2001
	5, 2.5, and 1.25 mg/kg, 10 mg/kg; intraperitoneal injection	Increase in long-term (300 days) animal survival (together with surgery), complete destruction of several DA3-derived metastatic foci in lungs (10 mg/kg, 72 h)	Blank et al., 2004
**CLINICAL STUDIES**			
Glioblastoma (35 patients) Anaplastic astrocytoma (7 patients)	0.05–0.50 mg/kg; oral administration	Stabilization or slight reduction of tumor volume (7 of 42 patients = 17%), partial clinical response (> 50% tumor reduction; 2 of 42 patients = 2%), mild adverse effects (photosensitivity, erythema, vomiting, diarrhea, etc.)	Couldwell et al., 2011

BrdU, 5-bromo-2′-deoxyuridine; MTT, 3-(4,5-dimethylthiazol-2-yl)-2,5-diphenyltetrazolium bromide; SCC, squamous cell carcinoma.

It has been found that non-activated hypericin possesses no cytotoxicity toward various cancer cell lines at concentrations sufficient for its photocytotoxic action (Thomas and Pardini, 1992; Hadjur et al., 1996; Vandenbogaerde et al., 1997). However, some *in vitro* studies have shown that hypericin can act as a cytotoxic or antiproliferative agent even in the dark (Blank et al., 2001, 2003; Berlanda et al., 2010; Besic Gyenge et al., 2012). The presence or absence of these effects often strongly depends on the hypericin concentration (higher concentrations are required than for HY-PDT-mediated toxicity), treatment conditions, the applied experimental methods, as well as on the type, origin and sensitivity of cancer cells. Moreover, most *in vitro* studies have tested the cytotoxicity of non-activated hypericin following its single dose or during relatively short time intervals; however, the situation after multiple fractionated hypericin dosages or during a long-term investigation might be completely different.

Firstly, Blank et al. (2001) demonstrated the cytotoxic and antiproliferative effect of non-activated hypericin both *in vitro* and *in vivo*. Hypericin significantly decreased the viability of highly metastatic murine breast adenocarcinoma (DA3^*HI*^) and SCC (SQ2) cells. Moreover, the anticancer potential of hypericin was observed even *in vivo* in DA3^*HI*^- and SQ2-derived tumors. Even though hypericin slightly accelerated death in mice with DA3^*HI*^-derived tumor development that was very rapid, intraperitoneal hypericin administration (6 declining doses) in other animals led to the inhibition of tumor growth, which was accompanied by prolonged survival time. Moreover, the hypericin-mediated improvement of survival was also evident in mice with high-grade SCC tumors (Blank et al., 2001). Subsequently, Blank et al. (2004) examined the antimetastatic potential of non-activated hypericin and evaluated its influence on long-term survival (up to 300 days). Again, mice with breast adenocarcinoma or SCC tumors, which develop metastases predominantly in the lungs, were used as experimental models. To evaluate the impact of hypericin only on metastases, primary tumors were surgically excised at a stage when micrometastases already existed. Hypericin was administered intraperitoneally in multiple declining dosages (up to 6 doses of hypericin at 5-day intervals). In both cases of tumor origin, hypericin therapy together with the resection of primary tumors resulted in a significant increase in long-term animal survival compared to the untreated control or to those animals that received surgery alone. Moreover, the complete destruction of several, but not all lung metastases, was evident 72 h after hypericin treatment. The results indicate that a single hypericin dose was insufficient to prolong the survival of animals, but fractionated hypericin doses could prevent animal death when administered shortly after the resection of the primary tumor (Blank et al., 2004). Similarly, multiple hypericin doses were applied in a previous study to obtain positive anticancer therapeutic results (Blank et al., 2001).

Considering the potential of hypericin to destroy metastases (Blank et al., 2004), the results are consistent with previous *in vitro* findings, where a decrease in the viability of DA-3^HI^ and SQ2 cells was also evident 72 h after treatment (Blank et al., 2001). However, no hypericin-mediated induction of apoptosis in the dark was observed. The inhibition of DNA synthesis indicated that the anticancer action of non-activated hypericin in these cell lines was more cytostatic than cytotoxic (Blank et al., 2001). Nevertheless, another *in vitro* experiment conducted by the same research group established mitotic cell death as the mechanism of hypericin-mediated cytotoxicity. Hypericin was responsible for the enhanced ubiquitinylation of the Hsp90 chaperone, resulting in the destabilization of its client proteins engaged in the regulation of cell proliferation, including p53, Cdk4, Plk, and Raf-1. Ultimately, cytostasis and a decrease in cell viability with no apoptosis were observed. Mitotic cell death is generally characterized by cell-cycle arrest in the G2/M phase, increased cell volume and multinucleation. All these phenotypes were evident in DA3 and SQ2 cells and even in B16.F10 melanoma cells after hypericin treatment (Blank et al., 2003). Thus, mitotic cell death might participate in the tumoricidal and antimetastatic functions of hypericin in the absence of light.

Martínez-Poveda et al. (2005) described another mechanism that might be implicated in the anticancer effects of hypericin in the dark. The results of several *in vitro* assays indicated that hypericin can inhibit several key steps of angiogenesis, including the proliferation, migration and invasion of endothelial cells, extracellular matrix-degrading urokinase or tubular formation on Matrigel (Martínez-Poveda et al., 2005). All these effects might be beneficial in the prevention of tumor neovascularization.

To our knowledge, one clinical trial has investigated the impact of hypericin on recurrent malignant gliomas (anaplastic astrocytoma and glioblastoma) and has monitored the tolerance of the patients to this treatment. Hypericin was administered orally at gradually increasing dosages ranging from 0.05 to 0.5 mg/kg (once each morning for up to 3 months) and was well-tolerated (mean maximum tolerated daily dose of 0.40 ± 0.098 mg/kg), although some mild skin or gastrointestinal side effects were observed. More importantly, hypericin stabilized or slightly reduced tumor volume (in seven out of 42 patients). In addition, a partial response (>50% reduction of tumor volume) was observed in two patients (Couldwell et al., 2011). These results indicate that synthetic orally administered hypericin can be moderately effective as an adjuvant therapy in cases of malignant glioma; however, further clinical studies are required.

### Hypericin as a necrosis-avid agent in oncology

In addition to its preferential accumulation in tumors compared to in normal healthy tissue, hypericin also has a specific strong affinity toward necrotic tissue (Van de Putte et al., 2008a,b,c). The mechanisms of this phenomenon have not yet been fully elucidated; although some hypotheses already exist that consider the binding of hypericin to specific constituents in the necrotic space. Several radiolabeled hypericin derivatives, particularly [^123^I]iodohypericin (Fonge et al., 2007) and [^131^I]iodohypericin (Li et al., 2011), possess similar necrosis avidity; thus, hypericin can be used as a potent necrosis-avid contrast agent (NACA) for the non-invasive detection and imaging of various necrosis-related pathologies and diseases or to assess tissue viability and therapeutic responses. Moreover, iodohypericins as NACAs might also be effective in relatively new approaches that combine both tumor diagnosis and therapy, so-called “theranostic” modalities (Table 6).

**Table 6:** **Preclinical *in vivo* studies to test hypericin as a necrosis-avid agent**.

**Experimental model/Type of tumor (cell line)**	**Hypericin derivate**	**Detection method**	**Effects**	**References**
C3H mice/Fibrosarcoma (RIF-1)	[^123^I]MIH	Gamma counter	Retention by the tumors and rapid clearance from healthy organs (faster clearance than [^123^I]MIprotoH)	Fonge et al., 2007
C3H/Km mice/Fibrosarcoma (RIF-1)	hypericin	FM imaging	Accumulation in intratumoral necrosis (4 h after administration)	Van de Putte et al., 2008a
		UV_365_ and Tungsten light, FM imaging	Preferential accumulation in intratumoral necrosis (intratumoral necrosis > viable tumor > normal liver tissue), enhanced contrast between necrotic and viable tissue = early assessment of therapeutic response (diagnosis)	Van de Putte et al., 2008b,c
Nude mice/Mammary cancer (BT474)	^64^CuBDH	PET, autoradiography	Higher accumulation in treated than in non-treated tumors = assessment of therapeutic response (diagnosis)	Song et al., 2011
Balb/c mice/Fibrosarcoma (RIF-1)	hypericin, [^123^I]MIH, [^131^I]MIH	FM, PET, autoradiography, scintigraphy	Longer persistence of tracers in necrotic than in viable tumor, stabilization of tumor growth and reduced tumor volume (3 injections of [^131^I]MIH) = potential in TNT	Van de Putte et al., 2012
WAG/Rij rats/Rhabdomyosarcoma (R1)	[^131^I]MIH	MRI, CT scan, scintigraphy, Gamma counter, autoradiography	Reduced tumor volume, prolonged tumor doubling time and inhibited tumor regrowth (dual-targeting with CA4P)	Li et al., 2011
	[^123^I]MIH, [^131^I]MIH		Accumulation in intratumoral necrosis ([^123^I]MIH); reduced tumor volume, prolonged tumor doubling time and increased intratumoral necrosis = tumoricidal effect (dual-targeting - [^131^I]MIH with CA4P)	Li et al., 2012
SCID mice/Fibrosarcoma (RIF-1)	[^131^I]MIH	MRI, scintiscan, autoradiography	Accumulation in intratumoral necrosis (over 120 h); prolonged survival time, marked radiation-induced cell death, reduced tumor volume, prolonged tumor doubling time (dual-targeting with CA4P)	Li et al., 2013
New Zealand white rabbits/VX2 tumors	[^131^I]MIH	MRI, SPECT, autoradiography	High targetability to tumor necrosis; reduced tumor growth and prolonged tumor doubling time (dual-targeting with CA4P)	Shao et al., 2015
Kunming (KM) mice/Hepatoma (H22) Sarcoma (S180)	[^131^I]MIH	FM, SPECT, autoradiography	Prolonged retention by the tumors, limited systemic toxicity, tumor growth delay = therapeutic efficacy	Liu et al., 2015

[^123^I]MIH, mono-[^123^I]iodohypericin; [^123^I]MIprotoH, mono-[^123^I]iodoprotohypericin; [^131^I]MIH, mono-[^131^I]iodohypericin; ^64^CuBDH, ^64^Cu-bis-DOTA-hypericin; CA4P, combretastatin A4 phosphate; CT, computed tomography; FM, fluorescence microscopy; MRI, magnetic resonance imaging; PET, positron emission tomography; SPECT, single-photon emission computed tomography; TNT, tumor necrosis therapy.

In a few initial *in vivo* studies, hypericin was investigated as a potential indicator of therapeutic responses, following various necrosis-inducing anticancer treatments. Mice bearing intrahepatic fibrosarcoma tumors were used as an experimental model and hypericin was injected intravenously 1 h before, or 24 h after intratumoral ethanol injection (Van de Putte et al., 2008b) or radiofrequency ablation (Van de Putte et al., 2008c), which both induced tumor necrosis. Fluoromacroscopic and fluoromicroscopic examinations confirmed that hypericin accumulated preferentially in necrotic tissue. In the cases of necrosis, mean fluorescence densities were about 4.5- and 5-fold higher than in viable tumor tissue and 14- and 12-fold higher than in normal liver tissue. These results demonstrate the ability of hypericin to enhance the imaging contrast between necrotic and viable tissues and ultimately, its potential role in the early assessment of the therapeutic response (Van de Putte et al., 2008b,c). At about the same time, tumor uptake of radiolabeled hypericin (mono-[^123^I]iodohypericin) and protohypericin (mono-[^123^I]iodoprotohypericin) derivatives were tested and compared, because of the applicability of tomographic imaging techniques instead of fluorescence-based techniques. Radioactivity was measured using a gamma counter. Both radiolabeled compounds were retained by the tumors, but mono-[^123^I]iodohypericin appeared to be a more suitable tumor diagnostic agent, due to its faster clearance from healthy organs (Fonge et al., 2007). Another radiolabeled derivate, ^64^Cu-bis-DOTA-hypericin, was also applicable in the early determination of the therapeutic response as its accumulation was significantly lower in non-treated tumors than in those treated by photothermal ablation therapy, inducing necrosis (Song et al., 2011).

As necrotic tissue represents 30–80% of the solid tumor mass and is rarely present in normal healthy tissue and organs, it is a suitable target not only for cancer diagnosis, but also for anticancer therapy. The strong avidity of iodohypericins for necrotic tissue makes these compounds very potent in the imaging of tumor necrosis. Furthermore, as hypericin can persist in necrotic tumor areas much longer (up to 72 h) than in viable tumor tissue (up to 24 h) (Van de Putte et al., 2012), its radiolabeled derivatives could be also used for so-called tumor necrosis therapy (TNT). This therapy is based on the destruction of adjacent viable tumor cells by the deposition and accumulation of radiation energy. In other words, attached radioactive iodine “bombards” the neighboring living tumor cells with radiation. Each successive treatment kills more tumor cells, thus, increasing the necrotic region, which allows higher efficacy with each treatment. As three injections of [^131^I]iodohypericin reduced the volume of RIF-1-derived tumors, this radiolabeled derivate might have potential in TNT (Van de Putte et al., 2012). In addition, similar results, especially the intense retention of [^131^I]iodohypericin in necrotic tumor tissue (over 168 h) and inhibited tumor growth after a single dose, were obtained by Liu et al. (2015) in mice bearing hepatomas or sarcomas.

Moreover, the approach of a necrosis-based anticancer treatment has been expanded into a dual-targeting theranostic strategy by administering the vascular-disrupting agent prior to the hypericin iododerivate. Firstly, a vascular-disrupting agent, such as combretastatin A4 phosphate (CA4P), targets the tumor microenvironment and subsequently, iodine radioactivity kills residual cancer cells. Several preclinical studies have demonstrated that dual-targeting using CA4P with [^131^I]iodohypericin was much more effective than single treatments. A reduced tumor volume, a prolonged tumor doubling time, an increase in radiation-induced cell death and intratumoral necrosis or prolonged survival time were reported in rats bearing liver rhabdomyosarcomas (Li et al., 2011, 2012), in mice bearing fibrosarcomas (Li et al., 2013) and in rabbits with liver and muscular VX2 tumors (Shao et al., 2015). Thus, the dual-targeting theranostic approach appears to be well tolerated and can enhance the therapeutic response, encouraging further development for other preclinical and even clinical applications.

### Non-activated hypericin and its potential negative impact on cancer treatment

All the above-mentioned preclinical and clinical results suggest that hypericin offers great potential in tumor diagnosis as well as in anticancer therapy. However, this secondary metabolite might also cause some other effects that would not be beneficial for therapeutic outcomes. It is well-known that the efficacy of commonly used anticancer treatment modalities is often limited by intrinsic or acquired MDR—a multifactorial phenomenon of the increased tolerance of cancer cells to various tumoricidal agents. A number of cellular mechanisms can contribute to MDR (reviewed in Stavrovskaya, 2000; Zahreddine and Borden, 2013), including the increased elimination of anticancer drugs by tumor cells, which is mostly linked to the elevated expression and/or activity of several ABC transporters. It has been shown that non-activated hypericin can modulate some of these efflux pumps. *In vitro* experiments conducted by our research group (Jendželovský et al., 2009; Jendželovská et al., 2014; Kuchárová et al., 2015) have revealed an increased expression of multidrug resistance-associated protein 1 (MRP1) and breast cancer resistance protein (BCRP) in colorectal HT-29 cells or in ovarian A2780 and A2780cis cells following hypericin treatment in the dark. In A2780 and A2780cis cells, 0.5 μM hypericin elevated MRP1 protein levels already 6 h after the treatment (Jendželovská et al., 2014). For HT-29 cells, an even lower hypericin concentration (0.1 μM) was sufficient to increase MRP1 and BCRP expression (16 h after hypericin addition) (Jendželovský et al., 2009; Kuchárová et al., 2015). Therefore, because many chemotherapeutic agents and photosensitizers are substrates of the above-mentioned transporters (reviewed in Nies et al., 2007; Robey et al., 2007), the hypericin-mediated stimulation of efflux systems might lead to a decrease in the efficacy of these therapeutic approaches when they are applied at the same time or shortly following hypericin treatment.

Wada et al. (2002) evaluated the impact of hypericin on the action of several anticancer drugs, using the cervical HeLa cell line and its resistant subline Hvr100-6 that overexpress another MDR-related ABC transporter, P-glycoprotein (P-gp), but no effect was observed. Several studies suggest that hypericin can neither modulate P-gp expression nor its activity (Wada et al., 2002; Tian et al., 2005; Jendželovský et al., 2009), which explains its poor ability to influence the cytotoxicity and transport of P-gp substrates, such as paclitaxel, daunorubicin, doxorubicin or vinblastin (Wada et al., 2002). On the other hand, 24 h pre-treatment with hypericin resulted in the attenuation of mitoxantrone cytotoxicity in HL-60 cells and cisplatin cytotoxicity in sensitive A2780 and resistant A2780cis cells (Jendželovská et al., 2014). However, this effect was probably not caused by modulation of the analyzed ABC transporters. However, the results suggest that hypericin in the dark might have a negative impact on the onset or progress of cell death induced by some anticancer agents, possibly by affecting some other mechanisms. Further studies are required, to elucidate the specific mechanisms responsible for the above-mentioned changes and *in vivo* studies will be necessary to verify the impact of non-activated hypericin on the outcome of chemotherapy.

Moreover, some MDR mechanisms, including ABC transporters, are also involved in drug pharmacokinetics. The modulation of these mechanisms can affect the absorption, distribution or clearance and ultimately, the action of the administered xenobiotics, resulting in negative drug interactions. Several clinical trials have demonstrated the interactions between some chemotherapeutic agents and St. John's wort extract, which is often taken by oncological patients as an antidepressant. In the first study, the enhanced metabolism of irinotecan and consequently, decreased plasma levels of its active metabolite (SN-38) were evaluated in cancer patients following St. John's wort treatment (Mathijssen et al., 2002). Furthermore, Frye et al. (2004) and Smith et al. (2004) examined the effect of St. John's wort extract on the pharmacokinetics of imatinib mesylate in healthy adult volunteers. In both studies, imatinib was administered before and after the treatment with St. John's wort (300 mg three times daily for 2 weeks) and its clearance and half-life was significantly increased or decreased, respectively, by the herb extract. Similar results were obtained by Goey et al. (2014) using a similar experimental design. Besides the enhanced clearance and decreased plasma concentrations of docetaxel in cancer patients, St. John's wort lowered the incidence of docetaxel-mediated toxicities. Thus, due to the risk of potential undertreatment, combining anticancer therapeutic approaches with St. John's wort extracts should not be recommended to oncological patients.

The probable reason for these effects is the induction of the metabolic enzyme (CYP3A4) and/or the P-gp transporter by hyperforin (Komoroski et al., 2004; Tian et al., 2005), another St. John's wort metabolite. However, considering the potential of hypericin to induce the expression of some ABC efflux pumps, this secondary metabolite might also contribute to negative drug interactions with St. John's wort. Therefore, a much broader spectrum of antineoplastic drugs might exist, including various chemotherapeutic agents or photosensitizers, whose action might be altered due to the presence of hypericin.

## Summary

In this review, we have summarized the medicinal applications of light-activated and non-activated hypericin in the field of oncology. We have preferentially highlighted the “primary beneficial side” of this secondary metabolite concerning its anticancer potential, but have also outlined its “second non-beneficial side,” which was revealed by preliminary *in vitro* studies.

The vast majority of the results summarized here suggest that hypericin in light and dark conditions might be a very potent agent in cancer treatment and diagnosis. Besides the well-known and intensively investigated HY-PDT and HY-PDD, some new and promising approaches using hypericin as NACA are becoming the focus of various research groups. Moreover, some tested modalities, including dual-targeting, might even improve the clinical outcome of cancer treatments. However, in the dark, hypericin might be responsible for the limited efficacy of conventionally applied chemotherapy or even PDT, due to its ability to induce the expression of some ABC transporters. Moreover, there is a suspicion that non-activated hypericin possesses much broader biological activity. Thus, the chronic usage of St. John's wort extracts as an antidepressant by oncological patients undergoing anticancer treatment should be avoided.

## Author contributions

ZJ designed the concept and issue of the review, studied the literature, contributed to all chapters and summarized the bulk of the text, revised the text after it was completed and also following English revision and approved the final version. RJ discussed the concept of the review with first author, studied the literature and contributed to the chapters about HY-PDT and non-activated hypericin, revised the text and approved the final version. BK studied the literature and contributed to the chapters about HY-PDT and non-activated hypericin, revised the text and approved the final version. PF discussed the concept of the review with first author, revised the text and approved the final version.

### Conflict of interest statement

The authors declare that the research was conducted in the absence of any commercial or financial relationships that could be construed as a potential conflict of interest.

## Abbreviations

^1^O_2_: singlet oxygen
^64^Cu-bis-DOTA-hypericin: ^64^Cu-Labeled bis-1, 4, 7, 10-tetraazacyclododecane-N,N′,N,N′*-tetraacetic acid conjugated hypericin*
ABC: ATP-binding cassette
AK: actinic keratosis
BCC: basal cell carcinoma
BCRP: breast cancer resistance protein
BD: Bowen's disease
CA4P: combretastatin A4 phosphate
Cdk4: cyclin-dependent kinase 4
CIS: carcinoma *in situ*
CLE: confocal laser endomicroscopy
CYP3A4: cytochrome P450 3A4
DAMPs: damage-associated molecular patterns
DLI: drug–light interval
EGFR: epidermal growth factor receptor
FE: fluorescence endoscopy
Hsp90: heat shock protein 90
HY-PDD: hypericin-mediated photodynamic diagnosis
HY-PDT: hypericin-mediated photodynamic therapy
ICD: immunogenic cell death
INF-α: interferon-α
LIF: laser-induced fluorescence
MDR: multidrug resistance
MF: mycosis fungoides
MRP1: multidrug resistance-associated protein 1
NACA: necrosis-avid contrast agent
${\text{O}}_{2}^{›-}$: superoxide anion
p53: phosphoprotein p53, tumor suppressor p53, tumor protein p53
PDD: photodynamic diagnosis
PDT: photodynamic therapy
P-gp: P-glycoprotein
Plk: Polo-like kinase
PVP: polyvinylpyrrolidone
Raf-1: serine/threonine kinase, Raf-1 proto-oncogene
ROS: reactive oxygen species
SCC: squamous cell carcinoma
Ser: serine
SN-38: 7-Ethyl-10-hydroxy-camptothecin
TCC: transitional cell carcinoma
Thr: threonine
TNF-α: tumor necrosis growth factor-α
TNT: tumor necrosis therapy
VEGF: vascular endothelial growth factor
WLE: white-light endoscopy.

## References

[B1] Abbaci M. Casiraghi O. Temam S. Ferchiou M. Bosq J. Dartigues P.. (2015). Red and far-red fluorescent dyes for the characterization of head and neck cancer at the cellular level. J. Oral Pathol. Med. 44, 831–841. 10.1111/jop.12316 25779631

[B2] Agostinis P. Berg K. Cengel K. A. Foster T. H. Girotti A. W. Gollnick S. O.. (2011). Photodynamic therapy of cancer: an update. CA Cancer J. Clin. 61, 250–281. 10.3322/caac.20114 21617154 PMC3209659

[B3] Agostinis P. Vantieghem A. Merlevede W. de Witte P. A. (2002). Hypericin in cancer treatment: more light on the way. Int. J. Biochem. Cell Biol. 34, 221–241. 10.1016/S1357-2725(01)00126-1 11849990

[B4] Alecu M. Ursaciuc C. Hãlãlãu F. Coman G. Merlevede W. Waelkens E.. (1998). Photodynamic treatment of basal cell carcinoma and squamous cell carcinoma with hypericin. Anticancer Res. 18, 4651–4654. 9891535

[B5] Ali S. M. Olivo M. (2002). Bio-distribution and subcellular localization of Hypericin and its role in PDT induced apoptosis in cancer cells. Int. J. Oncol. 21, 531–540. 10.3892/ijo.21.3.531 12168096

[B6] Ayan A. K. Cirak C. Kevseroglu K. Ozen T. (2004). Hypericin in some *Hypericum* species from Turkey. Asian J. Plant Sci. 3, 200–202. 10.3923/ajps.2004.200.202

[B7] Berlanda J. Kiesslich T. Engelhardt V. Krammer B. Plaetzer K. (2010). Comparative *in vitro* study on the characteristics of different photosensitizers employed in PDT. J. Photochem. Photobiol. B. Biol. 100, 173–180. 10.1016/j.jphotobiol.2010.06.004 20599390

[B8] Besic Gyenge E. Forny P. Lüscher D. Laass A. Walt H. Maake C. (2012). Effects of hypericin and a chlorin based photosensitizer alone or in combination in squamous cell carcinoma cells in the dark. Photodiagnosis Photodyn. Ther. 9, 321–331. 10.1016/j.pdpdt.2012.03.006 23200013

[B9] Bhuvaneswari R. Gan Y. Y. Lucky S. S. Chin W. W. Ali S. M. Soo K. C.. (2008). Molecular profiling of angiogenesis in hypericin mediated photodynamic therapy. Mol. Cancer. 7:56. 10.1186/1476-4598-7-56 18549507 PMC2440549

[B10] Bhuvaneswari R. Thong P. S. Gan Y. Y. Soo K. C. Olivo M. (2010). Evaluation of hypericin-mediated photodynamic therapy in combination with angiogenesis inhibitor bevacizumab using *in vivo* fluorescence confocal endomicroscopy. J. Biomed. Opt. 15:011114. 10.1117/1.3281671 20210440

[B11] Bhuvaneswari R. Yuen G. Y. Chee S. K. Olivo M. (2011). Antiangiogenesis agents avastin and erbitux enhance the efficacy of photodynamic therapy in a murine bladder tumor model. Lasers Surg. Med. 43, 651–662. 10.1002/lsm.21109 22057493

[B12] Blank M. Kostenich G. Lavie G. Kimel S. Keisari Y. Orenstein A. (2002). Wavelength-dependent properties of photodynamic therapy using hypericin *in vitro* and in an animal model. Photochem. Photobiol. 76, 335–340. 10.1562/0031-8655(2002)0760335WDPOPT2.0.CO2 12403456

[B13] Blank M. Lavie G. Mandel M. Hazan S. Orenstein A. Meruelo D.. (2004). Antimetastatic activity of the photodynamic agent hypericin in the dark. Int. J. Cancer. 111, 596–603. 10.1002/ijc.20285 15239139

[B14] Blank M. Mandel M. Hazan S. Keisari Y. Lavie G. (2001). Anti-cancer activities of hypericin in the dark. Photochem. Photobiol. 74, 120–125. 10.1562/0031-8655(2001)0740120ACAOHI2.0.CO2 11547544

[B15] Blank M. Mandel M. Keisari Y. Meruelo D. Lavie G. (2003). Enhanced ubiquitinylation of heat shock protein 90 as a potential mechanism for mitotic cell death in cancer cells induced with hypericin. Cancer Res. 63, 8241–8247. 14678981

[B16] Bobrov N. Cavarga I. Longauer F. Rybárová S. Fedorocko P. Brezáni P.. (2007). Histomorphological changes in murine fibrosarcoma after hypericin-based photodynamic therapy. Phytomedicine 14, 172–178. 10.1016/j.phymed.2006.09.017 17095201

[B17] Boiy A. Roelandts R. de Witte P. A. (2010). Photodynamic therapy using topically applied hypericin: comparative effect with methyl-aminolevulinic acid on UV induced skin tumours. J. Photochem. Photobiol. B. Biol. 102, 123–131. 10.1016/j.jphotobiol.2010.09.012 21035351

[B18] Brockmann H. Haschad M. N. Maier K. Pohl F. (1939). Über das Hypericin, den photodynamisch wirksamen Farbstoff aus *Hypericum perforatum*. Naturwissenschaften 27, 550 10.1007/BF01495453

[B19] Bruňáková K. Petijová L. Zámečník J. Turečková V. Čellárová E. (2015). The role of ABA in the freezing injury avoidance in two *Hypericum* species differing in frost tolerance and potential to synthesize hypericins. Plant Cell Tissue Organ Cult. 122, 45–56. 10.1007/s11240-015-0748-9

[B20] Buytaert E. Callewaert G. Hendrickx N. Scorrano L. Hartmann D. Missiaen L.. (2006). Role of endoplasmic reticulum depletion and multidomain proapoptotic BAX and BAK proteins in shaping cell death after hypericin-mediated photodynamic therapy. FASEB J. 20, 756–758. 10.1096/fj.05-4305fje 16455754

[B21] Čavarga I. Brezáni P. Cekanová-Figurová M. Solár P. Fedorocko P. Miskovský P. (2001). Photodynamic therapy of murine fibrosarcoma with topical and systemic administration of hypericin. Phytomedicine 8, 325–330. 10.1078/0944-7113-00057 11695874

[B22] Čavarga I. Brezáni P. Fedorocko P. Miskovský P. Bobrov N. Longauer F.. (2005). Photoinduced antitumour effect of hypericin can be enhanced by fractionated dosing. Phytomedicine 12, 680–683. 10.1016/j.phymed.2004.02.011 16194057

[B23] Čellárová E. (2011). Effect of exogenous morphogenetic signals on differentiation *in vitro* and secondary metabolite formation in the genus *Hypericum* in Medicinal and Aromatic Plant Science and Biotechnology 5 (Special Issue 1), eds Odabas M. S. Çırak C. (Ikenobe: Global Science Books), 62–69.

[B24] Čellárová E. Brutovská R. Daxnerová Z. Bruňáková K. Weigel R. C. (1997). Correlation between hypericin content and the ploidy of somaclones of *Hypericum perforatum* L. Acta Biotechnol. 17, 83–90. 10.1002/abio.370170111

[B25] Chen B. de Witte P. A. (2000). Photodynamic therapy efficacy and tissue distribution of hypericin in a mouse P388 lymphoma tumor model. Cancer Lett. 150, 111–117. 10.1016/S0304-3835(99)00381-X 10755394

[B26] Chen B. Roskams T. de Witte P. A. (2002a). Antivascular tumor eradication by hypericin-mediated photodynamic therapy. Photochem. Photobiol. 76, 509–513. 10.1562/0031-8655(2002)0760509ATEBHM2.0.CO2 12462645

[B27] Chen B. Roskams T. Xu Y. Agostinis P. de Witte P. A. (2002b). Photodynamic therapy with hypericin induces vascular damage and apoptosis in the RIF-1 mouse tumor model. Int. J. Cancer. 98, 284–290. 10.1002/ijc.10175 11857421

[B28] Chen B. Xu Y. Roskams T. Delaey E. Agostinis P. Vandenheede J. R.. (2001). Efficacy of antitumoral photodynamic therapy with hypericin: relationship between biodistribution and photodynamic effects in the RIF-1 mouse tumor model. Int. J. Cancer. 93, 275–282. 10.1002/ijc.1324 11410877

[B29] Cole C. D. Liu J. K. Sheng X. Chin S. S. Schmidt M. H. Weiss M. H.. (2008). Hypericin-mediated photodynamic therapy of pituitary tumors: preclinical study in a GH4C1 rat tumor model. J. Neurooncol. 87, 255–261. 10.1007/s11060-007-9514-0 18228116

[B30] Couldwell W. T. Surnock A. A. Tobia A. J. Cabana B. E. Stillerman C. B. Forsyth P. A.. (2011). A phase 1/2 study of orally administered synthetic hypericin for treatment of recurrent malignant gliomas. Cancer 117, 4905–4915. 10.1002/cncr.26123 21456013

[B31] Crnolatac I. Huygens A. van Aerschot A. Busson R. Rozenski J. de Witte P. A. (2005). Synthesis, *in vitro* cellular uptake and photo-induced antiproliferative effects of lipophilic hypericin acid derivatives. Bioorg. Med. Chem. 13, 6347–6353. 10.1016/j.bmc.2005.09.003 16213734

[B32] Delaey E. Vandenbogaerde A. Merlevede W. de Witte P. (2000). Photocytotoxicity of hypericin in normoxic and hypoxic conditions. J. Photochem. Photobiol. B. Biol. 56, 19–24. 10.1016/S1011-1344(00)00051-8 11073312

[B33] Dewick P. M. (2002). Medicinal Natural Products: A Biosynthetic Approach, 2nd Edn. Chichester: John Wiley & Sons Ltd.

[B34] D'Hallewin M. A. De Witte P. A. Waelkens E. Merlevede W. Baert L. (2000). Fluorescence detection of flat bladder carcinoma *in situ* after intravesical instillation of hypericin. J. Urol. 164, 349–351. 10.1016/S0022-5347(05)67357-0 10893582

[B35] D'Hallewin M. A. Kamuhabwa A. R. Roskams T. De Witte P. A. Baert L. (2002). Hypericin-based fluorescence diagnosis of bladder carcinoma. BJU Int. 89, 760–763. 10.1046/j.1464-410X.2002.02690.x 11966641

[B36] Diwu Z. Lown J. W. (1993). Photosensitization with anticancer agents. 17. EPR studies of photodynamic action of hypericin: formation of semiquinone radical and activated oxygen species on illumination. Free Radic. Biol. Med. 14, 209–215. 10.1016/0891-5849(93)90012-J 8381107

[B37] Du H. Y. Bay B. H. Olivo M. (2003a). Biodistribution and photodynamic therapy with hypericin in a human NPC murine tumor model. Int. J. Oncol. 22, 1019–1024. 10.3892/ijo.22.5.1019 12684667

[B38] Du H. Y. Olivo M. Tan B. K. Bay B. H. (2003b). Hypericin-mediated photodynamic therapy induces lipid peroxidation and necrosis in nasopharyngeal cancer. Int. J. Oncol. 23, 1401–1405. 10.3892/ijo.23.5.1401 14532982

[B39] Feruszová J. Imreová P. Bodnárová K. Ševčovičová A. Kyzek S. Chalupa I. (2016). Photoactivated hypericin is not genotoxic. Gen. Physiol. Biophys. 35, 223–230. 10.4149/gpb_2015045 26891274

[B40] Fonge H. Van de Putte M. Huyghe D. Bormans G. Ni Y. de Witte P.. (2007). Evaluation of tumor affinity of mono-[(123)I]iodohypericin and mono-[(123)I]iodoprotohypericin in a mouse model with a RIF-1 tumor. Contrast Media Mol. Imaging. 2, 113–119. 10.1002/cmmi.136 17546702

[B41] Fonge H. Vunckx K. Wang H. Feng Y. Mortelmans L. Nuyts J.. (2008). Non-invasive detection and quantification of acute myocardial infarction in rabbits using mono-[123I]iodohypericin microSPECT. Eur. Heart J. 29, 260–269. 10.1093/eurheartj/ehm588 18156139

[B42] Frye R. F. Fitzgerald S. M. Lagattuta T. F. Hruska M. W. Egorin M. J. (2004). Effect of St John's wort on imatinib mesylate pharmacokinetics. Clin. Pharmacol. Ther. 76, 323–329. 10.1016/j.clpt.2004.06.007 15470331

[B43] Fu C. Y. Ng B. K. Razul S. G. Chin W. W. Tan P. H. Lau W. K.. (2007). Fluorescence detection of bladder cancer using urine cytology. Int. J. Oncol. 31, 525–530. 10.3892/ijo.31.3.525 17671678

[B44] Galanou M. C. Theodossiou T. A. Tsiourvas D. Sideratou Z. Paleos C. M. (2008). Interactive transport, subcellular relocation and enhanced phototoxicity of hypericin encapsulated in guanidinylated liposomes via molecular recognition. Photochem. Photobiol. 84, 1073–1083. 10.1111/j.1751-1097.2008.00392.x 18627515

[B45] Galluzzi L. Vacchelli E. Bravo-San Pedro J. M. Buqué A. Senovilla L. Baracco E. E.. (2014). Classification of current anticancer immunotherapies. Oncotarget 5, 12472–12508. 10.18632/oncotarget.2998 25537519 PMC4350348

[B46] García I. Ballesta S. Gilaberte Y. Rezusta A. Pascual Á. (2015). Antimicrobial photodynamic activity of hypericin against methicillin-susceptible and resistant *Staphylococcus aureus* biofilms. Future Microbiol. 10, 347–356. 10.2217/fmb.14.114 25812458

[B47] Garg A. D. Agostinis P. (2014). ER stress, autophagy and immunogenic cell death in photodynamic therapy-induced anti-cancer immune responses. Photochem. Photobiol. Sci. 13, 474–487. 10.1039/c3pp50333j 24493131

[B48] Garg A. D. Elsen S. Krysko D. V. Vandenabeele P. de Witte P. Agostinis P. (2015b). Resistance to anticancer vaccination effect is controlled by a cancer cell-autonomous phenotype that disrupts immunogenic phagocytic removal. Oncotarget 6, 26841–26860. 10.18632/oncotarget.4754 26314964 PMC4694957

[B49] Garg A. D. Galluzzi L. Apetoh L. Baert T. Birge R. B. Bravo-San Pedro J. M.. (2015a). Molecular and translational classifications of DAMPs in immunogenic cell death. Front. Immunol. 6:588. 10.3389/fimmu.2015.00588 26635802 PMC4653610

[B50] Garg A. D. Krysko D. V. Vandenabeele P. Agostinis P. (2012b). Hypericin-based photodynamic therapy induces surface exposure of damage-associated molecular patterns like HSP70 and calreticulin. Cancer Immunol. Immunother. 61, 215–221. 10.1007/s00262-011-1184-2 22193987 PMC11029694

[B51] Garg A. D. Krysko D. V. Vandenabeele P. Agostinis P. (2016). Extracellular ATP and P_2_X_7_ receptor exert context-specific immunogenic effects after immunogenic cancer cell death. Cell Death Dis. 7, e2097. 10.1038/cddis.2015.411 26890136 PMC5399185

[B52] Garg A. D. Krysko D. V. Verfaillie T. Kaczmarek A. Ferreira G. B. Marysael T.. (2012a). A novel pathway combining calreticulin exposure and ATP secretion in immunogenic cancer cell death. EMBO J. 31, 1062–1079. 10.1038/emboj.2011.497 22252128 PMC3298003

[B53] Garnica S. Weiss M. Oberwinkler F. (2003). Morphological and molecular phylogenetic studies in South American Cortinarius species. Mycol. Res. 107, 1143–1156. 10.1017/S0953756203008414 14635763

[B54] Goey A. K. Meijerman I. Rosing H. Marchetti S. Mergui-Roelvink M. Keessen M.. (2014). The effect of St John's wort on the pharmacokinetics of docetaxel. Clin. Pharmacokinet. 53, 103–110. 10.1007/s40262-013-0102-5 24068654

[B55] Gulick R. M. McAuliffe V. Holden-Wiltse J. Crumpacker C. Liebes L. Stein D. S.. (1999). Phase I studies of hypericin, the active compound in St. John's Wort, as an antiretroviral agent in HIV-infected adults. AIDS Clinical Trials Group Protocols 150 and 258. Ann Intern Med. 130, 510–514. 10.7326/0003-4819-130-6-199903160-00015 10075619

[B56] Hadjur C. Richard M. J. Parat M. O. Jardon P. Favier A. (1996). Photodynamic effects of hypericin on lipid peroxidation and antioxidant status in melanoma cells. Photochem. Photobiol. 64, 375–381. 10.1111/j.1751-1097.1996.tb02474.x 8760577

[B57] Head C. S. Luu Q. Sercarz J. Saxton R. (2006). Photodynamic therapy and tumor imaging of hypericin-treated squamous cell carcinoma. World J. Surg. Oncol. 4:87. 10.1186/1477-7819-4-87 17147827 PMC1762016

[B58] Hudson J. B. Harris L. Towers G. H. (1993). The importance of light in the anti-HIV effect of hypericin. Antiviral Res. 20, 173–178. 10.1016/0166-3542(93)90006-5 8460933

[B59] Huygens A. Kamuhabwa A. R. Van Laethem A. Roskams T. Van Cleynenbreugel B. Van Poppel H.. (2005). Enhancing the photodynamic effect of hypericin in tumour spheroids by fractionated light delivery in combination with hyperoxygenation. Int. J. Oncol. 26, 1691–1697. 10.3892/ijo.26.6.1691 15870887

[B60] Jacobson J. M. Feinman L. Liebes L. Ostrow N. Koslowski V. Tobia A.. (2001). Pharmacokinetics, safety, and antiviral effects of hypericin, a derivative of St. John's wort plant, in patients with chronic hepatitis C virus infection. Antimicrob. Agents Chemother. 45, 517–524. 10.1128/AAC.45.2.517-524.2001 11158749 PMC90321

[B61] Jendželovská Z. Jendželovský R. Hil'ovská L. Koval' J. Mikeš J. Fedoročko P. (2014). Single pre-treatment with hypericin, a St. John's wort secondary metabolite, attenuates cisplatin- and mitoxantrone-induced cell death in A2780, A2780cis and HL-60 cells. Toxicol. In vitro 28, 1259–1273. 10.1016/j.tiv.2014.06.011 24994473

[B62] Jendželovský R. Mikes J. Koval,' J. Soucek K. Procházková J. Kello M.. (2009). Drug efflux transporters, MRP1 and BCRP, affect the outcome of hypericin-mediated photodynamic therapy in HT-29 adenocarcinoma cells. Photochem. Photobiol. Sci. 8, 1716–1723. 10.1039/b9pp00086k 20024169

[B63] Kacerovská D. Pizinger K. Majer F. Smíd F. (2008). Photodynamic therapy of nonmelanoma skin cancer with topical *Hypericum perforatum* extract–a pilot study. Photochem. Photobiol. 84, 779–785. 10.1111/j.1751-1097.2007.00260.x 18179625

[B64] Kamuhabwa A. A. Cosserat-Gerardin I. Didelon J. Notter D. Guillemin F. Roskams T.. (2002). Biodistribution of hypericin in orthotopic transitional cell carcinoma bladder tumors: implication for whole bladder wall photodynamic therapy. Int. J. Cancer. 97, 253–260. 10.1002/ijc.1594 11774272

[B65] Kamuhabwa A. A. Di Mavungu J. D. Baert L. D'Hallewin M. A. Hoogmartens J. de Witte P. A. (2005). Determination of hypericin in human plasma by high-performance liquid chromatography after intravesical administration in patients with transitional cell carcinoma of the bladder. Eur. J. Pharm. Biopharm. 59, 469–474. 10.1016/j.ejpb.2004.09.013 15760727

[B66] Kamuhabwa A. A. Roskams T. D'Hallewin M. A. Baert L. Van Poppel H. de Witte P. A. (2003). Whole bladder wall photodynamic therapy of transitional cell carcinoma rat bladder tumors using intravesically administered hypericin. Int. J. Cancer. 107, 460–467. 10.1002/ijc.11396 14506748

[B67] Kascakova S. Nadova Z. Mateasik A. Mikes J. Huntosova V. Refregiers M.. (2008). High level of low-density lipoprotein receptors enhance hypericin uptake by U-87 MG cells in the presence of LDL. Photochem. Photobiol. 84, 120–127. 10.1111/j.1751-1097.2007.00207.x 18173711

[B68] Kashef N. Borghei Y. S. Djavid G. E. (2013). Photodynamic effect of hypericin on the microorganisms and primary human fibroblasts. Photodiagnosis Photodyn. Ther. 10, 150–155. 10.1016/j.pdpdt.2012.11.007 23769281

[B69] Kasper S. Caraci F. Forti B. Drago F. Aguglia E. (2010). Efficacy and tolerability of *Hypericum* extract for the treatment of mild to moderate depression. Eur. Neuropsychopharmacol. 20, 747–765. 10.1016/j.euroneuro.2010.07.005 20708905

[B70] Kim M. Jung H. Y. Park H. J. (2015). Topical PDT in the treatment of benign skin diseases: principles and new applications. Int. J. Mol. Sci. 16, 23259–23278. 10.3390/ijms161023259 26404243 PMC4632697

[B71] Kitanov G. M. (2001). Hypericin and pseudohypericin in some *Hypericum* species. Biochem. Syst. Ecol. 29, 171–178. 10.1016/S0305-1978(00)00032-6 11106845

[B72] Kleemann B. Loos B. Scriba T. J. Lang D. Davids L. M. (2014). St John's Wort (*Hypericum perforatum L.*) photomedicine: hypericin-photodynamic therapy induces metastatic melanoma cell death. PLoS ONE 9:e103762. 10.1371/journal.pone.0103762 25076130 PMC4116257

[B73] Komoroski B. J. Zhang S. Cai H. Hutzler J. M. Frye R. Tracy T. S.. (2004). Induction and inhibition of cytochromes P450 by the St. John's wort constituent hyperforin in human hepatocyte cultures. Drug Metab. Dispos. 32, 512–518. 10.1124/dmd.32.5.512 15100173

[B74] Koperdáková J. Komarovská H. Košuth J. Giovannini A. Čellárová E. (2009). Characterization of hairy root-phenotype in transgenic *Hypericum perforatum* L. clones. Acta Physiol. Plant. 31, 351–358. 10.1007/s11738-008-0241-8

[B75] Košuth J. Koperdáková J. Tolonen A. Hohtola A. Cellárová E. (2003). The content of hypericins and phloroglucinols in *Hypericum perforatum* L. seedlings at early stage of development. Plant Sci. 165, 515–521. 10.1016/S0168-9452(03)00210-3

[B76] Koval J. Mikes J. Jendzelovský R. Kello M. Solár P. Fedorocko P. (2010). Degradation of HER2 receptor through hypericin-mediated photodynamic therapy. Photochem. Photobiol. 86, 200–205. 10.1111/j.1751-1097.2009.00639.x 19912559

[B77] Kubin A. Meissner P. Wierrani F. Burner U. Bodenteich A. Pytel A.. (2008). Fluorescence diagnosis of bladder cancer with new water soluble hypericin bound to polyvinylpyrrolidone: PVP-hypericin. Photochem. Photobiol. 84, 1560–1563. 10.1111/j.1751-1097.2008.00384.x 18627521

[B78] Kubin A. Wierrani F. Burner U. Alth G. Grünberger W. (2005). Hypericin–the facts about a controversial agent. Curr. Pharm. Des. 11, 233–253. 10.2174/1381612053382287 15638760

[B79] Kucharikova A. Kimakova K. Janfelt C. Čellarova E. (2016). Interspecific variation in localization of hypericins and phloroglucinols in the genus *Hypericum* as revealed by Desorption Electrospray Ionization Mass Spectrometry imaging. Physiol Plant. 157, 2–12. 10.1111/ppl.12422 26822391

[B80] Kuchárová B. Mikeš J. Jendželovský R. Vargová J. Mikešová L. Jendželovská Z.. (2015). Potentiation of hypericin-mediated photodynamic therapy cytotoxicity by MK-886: focus on ABC transporters, GDF-15 and redox status. Photodiagnosis Photodyn. Ther. 12, 490–503. 10.1016/j.pdpdt.2015.04.008 26003114

[B81] Kusari S. Lamshöft M. Zühlke S. Spiteller M. (2008). An endophytic fungus from *Hypericum perforatum* that produces hypericin. J. Nat. Prod. 71, 159–162. 10.1021/np070669k 18220354

[B82] Kusari S. Sezgin S. Nigutová K. Čellárová E. Spiteller M. (2015). Spatial chemo-profiling of hypericin and related phytochemicals in *Hypericum* species using MALDI-HRMS imaging. Anal. Bioanal. Chem. 407, 4779–4791. 10.1007/s00216-015-8682-6 25912460

[B83] Kusari S. Zühlke S. Košuth J. Čellárová E. Spiteller M. (2009). Light-independent metabolomics of endophytic *Thielavia subthermophila* provides insight into microbial hypericin biosynthesis. J. Nat. Prod. 72, 1825–1835. 10.1021/np9002977 19746917

[B84] Li J. Cona M. M. Chen F. Feng Y. Zhou L. Yu J.. (2012). Exploring theranostic potentials of radioiodinated hypericin in rodent necrosis models. Theranostics 2, 1010–1019. 10.7150/thno.4924 23139728 PMC3493203

[B85] Li J. Cona M. M. Chen F. Feng Y. Zhou L. Zhang G.. (2013). Sequential systemic administrations of combretastatin A4 Phosphate and radioiodinated hypericin exert synergistic targeted theranostic effects with prolonged survival on SCID mice carrying bifocal tumor xenografts. Theranostics 3, 127–137. 10.7150/thno.5790 23423247 PMC3575593

[B86] Li J. Sun Z. Zhang J. Shao H. Cona M. M. Wang H.. (2011). A dual-targeting anticancer approach: soil and seed principle. Radiology 260, 799–807. 10.1148/radiol.11102120 21712473

[B87] Liu C. D. Kwan D. Saxton R. E. McFadden D. W. (2000). Hypericin and photodynamic therapy decreases human pancreatic cancer *in vitro* and *in vivo*. J. Surg. Res. 93, 137–143. 10.1006/jsre.2000.5949 10945955

[B88] Liu W. Zhang D. Feng Y. Li Y. Huang D. Jiang C.. (2015). Biodistribution and anti-tumor efficacy of intratumorally injected necrosis-avid theranostic agent radioiodinated hypericin in rodent tumor models. J. Drug Target. 23, 371–379. 10.3109/1061186X.2014.1000337 25572455

[B89] Lukšienė Ž. De Witte P. (2002). Hypericin-based photodynamic therapy: I. Comparative antitumor activity uptake studies in Ehrlich ascite tumor. Acta Med Litu. 9, 195–199.

[B90] Martínez-Poveda B. Quesada A. R. Medina M. A. (2005). Hypericin in the dark inhibits key steps of angiogenesis *in vitro*. Eur. J. Pharmacol. 516, 97–103. 10.1016/j.ejphar.2005.03.047 15921677

[B91] Mathijssen R. H. Verweij J. de Bruijn P. Loos W. J. Sparreboom A. (2002). Effects of St. John's wort on irinotecan metabolism. J. Natl. Cancer Inst. 94, 1247–1249. 10.1093/jnci/94.16.1247 12189228

[B92] Miadokova E. Chalupa I. Vlckova V. Sevcovicova A. Nadova S. Kopaskova M.. (2010). Genotoxicity and antigenotoxicity evaluation of non-photoactivated hypericin. Phytother. Res. 24, 90–95. 10.1002/ptr.2901 19585477

[B93] Mikeš J. Hýžd'alová M. Kočí L. Jendželovský R. Koval' J. Vaculová A.. (2011). Lower sensitivity of FHC fetal colon epithelial cells to photodynamic therapy compared to HT-29 colon adenocarcinoma cells despite higher intracellular accumulation of hypericin. Photochem. Photobiol. Sci. 10, 626–632. 10.1039/c0pp00359j 21298151

[B94] Mikeš J. Jendželovský R. Fedoročko P. (2013). Cellular aspects of photodynamic therapy with hypericin in Photodynamic Therapy: New Research, ed Elsaie M. L. T. (New York, NY: Nova Science Publishers), 111–147.

[B95] Mikeš J. Kleban J. Sacková V. Horváth V. Jamborová E. Vaculová A.. (2007). Necrosis predominates in the cell death of human colon adenocarcinoma HT-29 cells treated under variable conditions of photodynamic therapy with hypericin. Photochem. Photobiol. Sci. 6, 758–766. 10.1039/B700350A 17609769

[B96] Mikeš J. Koval' J. Jendzelovský R. Sacková V. Uhrinová I. Kello M.. (2009). The role of p53 in the efficiency of photodynamic therapy with hypericin and subsequent long-term survival of colon cancer cells. Photochem. Photobiol. Sci. 8, 1558–1567. 10.1039/b9pp00021f 19862414

[B97] Mikešová L. Mikeš J. Koval' J. Gyurászová K. Culka L. Vargová J.. (2013). Conjunction of glutathione level, NAD(P)H/FAD redox status and hypericin content as a potential factor affecting colon cancer cell resistance to photodynamic therapy with hypericin. Photodiagnosis Photodyn. Ther. 10, 470–483. 10.1016/j.pdpdt.2013.04.003 24284100

[B98] Ni Y. Huyghe D. Verbeke K. de Witte P. A. Nuyts J. Mortelmans L.. (2006). First preclinical evaluation of mono-[123I]iodohypericin as a necrosis-avid tracer agent. Eur. J. Nucl. Med. Mol. Imaging 33, 595–601. 10.1007/s00259-005-0013-2 16450141

[B99] Nies A. T. Rius M. Keppler D. (2007). Multidrug resistance proteins of the ABCC subfamily in Drug Transporters: Molecular Characterization and Role in Drug Disposition, eds You G. Morris M. E. (New Jersey, NJ: John Wiley & Sons, Inc.), 223–262.

[B100] Noell S. Feigl G. C. Serifi D. Mayer D. Naumann U. Göbel W.. (2013). Microendoscopy for hypericin fluorescence tumor diagnosis in a subcutaneous glioma mouse model. Photodiagnosis Photodyn. Ther. 10, 552–560. 10.1016/j.pdpdt.2013.06.001 24284111

[B101] Noell S. Mayer D. Strauss W. S. Tatagiba M. S. Ritz R. (2011). Selective enrichment of hypericin in malignant glioma: pioneering *in vivo* results. Int. J. Oncol. 38, 1343–1348. 10.3892/ijo.2011.968 21399870

[B102] Olivo M. Lau W. Manivasager V. Bhuvaneswari R. Wei Z. Soo K. C.. (2003a). Novel photodynamic diagnosis of bladder cancer: *ex vivo* fluorescence cytology using hypericin. Int. J. Oncol. 23, 1501–1504. 10.3892/ijo.23.6.1501 14612919

[B103] Olivo M. Lau W. Manivasager V. Tan P. H. Soo K. C. Cheng C. (2003b). Macro-microscopic fluorescence of human bladder cancer using hypericin fluorescence cystoscopy and laser confocal microscopy. Int. J. Oncol. 23, 983–990. 10.3892/ijo.23.4.983 12963977

[B104] Paz-Cristobal M. P. Gilaberte Y. Alejandre C. Pardo J. Revillo M. J. Rezusta A. (2014). *In vitro* fungicidal photodynamic effect of hypericin on *Trichophyton* spp. Mycopathologia 178, 221–225. 10.1007/s11046-014-9797-6 25129421

[B105] Prince A. M. Pascual D. Meruelo D. Liebes L. Mazur Y. Dubovi E.. (2000). Strategies for evaluation of enveloped virus inactivation in red cell concentrates using hypericin. Photochem. Photobiol. 71, 188–195. 10.1562/0031-8655(2000)0710188SFEOEV2.0.CO2 10687393

[B106] Pytel A. Schmeller N. (2002). New aspect of photodynamic diagnosis of bladder tumors: fluorescence cytology. Urology 59, 216–219. 10.1016/S0090-4295(01)01528-X 11834388

[B107] Rezusta A. López-Chicón P. Paz-Cristobal M. P. Alemany-Ribes M. Royo-Díez D. Agut M.. (2012). *In vitro* fungicidal photodynamic effect of hypericin on Candida species. Photochem. Photobiol. 88, 613–619. 10.1111/j.1751-1097.2011.01053.x 22128758

[B108] Ritz R. Daniels R. Noell S. Feigl G. C. Schmidt V. Bornemann A.. (2012). Hypericin for visualization of high grade gliomas: first clinical experience. Eur. J. Surg. Oncol. 38, 352–360. 10.1016/j.ejso.2011.12.021 22284346

[B109] Robey R. W. Polgar O. Deeken J. To K. K. W. Bates S. (2007). Breast cancer resistance protein, in Drug Transporters: Molecular Characterization and Role in Drug Disposition, eds You G. Morris M. E. (New Jersey, NJ: John Wiley & Sons, Inc.), 319–358. 10.1002/9780470140505.ch12

[B110] Rook A. H. Wood G. S. Duvic M. Vonderheid E. C. Tobia A. Cabana B. (2010). A phase II placebo-controlled study of photodynamic therapy with topical hypericin and visible light irradiation in the treatment of cutaneous T-cell lymphoma and psoriasis. J. Am. Acad. Dermatol. 63, 984–990. 10.1016/j.jaad.2010.02.039 20889234

[B111] Rubio N. Coupienne I. Di Valentin E. Heirman I. Grooten J. Piette J.. (2012). Spatiotemporal autophagic degradation of oxidatively damaged organelles after photodynamic stress is amplified by mitochondrial reactive oxygen species. Autophagy 8, 1312–1324. 10.4161/auto.20763 22889744 PMC3442878

[B112] Sacková V. Fedorocko P. Szilárdiová B. Mikes J. Kleban J. (2006). Hypericin-induced photocytotoxicity is connected with G2/M arrest in HT-29 and S-phase arrest in U937 cells. Photochem. Photobiol. 82, 1285–1291. 10.1562/2006-02-22-RA-806 16740057

[B113] Sanovic R. Verwanger T. Hartl A. Krammer B. (2011). Low dose hypericin-PDT induces complete tumor regression in BALB/c mice bearing CT26 colon carcinoma. Photodiagnosis Photodyn. Ther. 8, 291–296. 10.1016/j.pdpdt.2011.04.003 22122915

[B114] Sattler S. Schaefer U. Schneider W. Hoelzl J. Lehr C. M. (1997). Binding, uptake, and transport of hypericin by Caco-2 cell monolayers. J. Pharm. Sci. 86, 1120–1126. 10.1021/js970004a 9344168

[B115] Shao H. Zhang J. Sun Z. Chen F. Dai X. Li Y.. (2015). Necrosis targeted radiotherapy with iodine-131-labeled hypericin to improve anticancer efficacy of vascular disrupting treatment in rabbit VX2 tumor models. Oncotarget 6, 14247–14259. 10.18632/oncotarget.3679 26036625 PMC4546464

[B116] Siboni G. Weitman H. Freeman D. Mazur Y. Malik Z. Ehrenberg B. (2002). The correlation between hydrophilicity of hypericins and helianthrone: internalization mechanisms, subcellular distribution and photodynamic action in colon carcinoma cells. Photochem. Photobiol. Sci. 1, 483–491. 10.1039/b202884k 12659159

[B117] Sim H. G. Lau W. K. Olivo M. Tan P. H. Cheng C. W. (2005). Is photodynamic diagnosis using hypericin better than white-light cystoscopy for detecting superficial bladder carcinoma? BJU Int. 95, 1215–1218. 10.1111/j.1464-410X.2005.05508.x 15892804

[B118] Smith P. Bullock J. M. Booker B. M. Haas C. E. Berenson C. S. Jusko W. J. (2004). The influence of St. John's wort on the pharmacokinetics and protein binding of imatinib mesylate. Pharmacotherapy 24, 1508–1514. 10.1592/phco.24.16.1508.50958 15537555

[B119] Song S. Xiong C. Zhou M. Lu W. Huang Q. Ku G.. (2011). Small-animal PET of tumor damage induced by photothermal ablation with 64Cu-bis-DOTA-hypericin. J. Nucl. Med. 52, 792–799. 10.2967/jnumed.110.086116 21498539

[B120] Stavrovskaya A. A. (2000). Cellular mechanisms of multidrug resistance of tumor cells. Biochemistry Mosc. 65, 95–106. 10702644

[B121] Straub M. Russ D. Horn T. Gschwend J. E. Abrahamsberg C. (2015). A phase IIA dose-finding study of PVP-hypericin fluorescence cystoscopy for detection of nonmuscle-invasive bladder cancer. J. Endourol. 29, 216–222. 10.1089/end.2014.0282 25222735

[B122] Theodossiou T. A. Hothersall J. S. De Witte P. A. Pantos A. Agostinis P. (2009). The multifaceted photocytotoxic profile of hypericin. Mol. Pharm. 6, 1775–1789. 10.1021/mp900166q 19739671

[B123] Thomas C. MacGill R. S. Miller G. C. Pardini R. S. (1992). Photoactivation of hypericin generates singlet oxygen in mitochondria and inhibits succinoxidase. Photochem. Photobiol. 55, 47–53. 10.1111/j.1751-1097.1992.tb04208.x 1603850

[B124] Thomas C. Pardini R. S. (1992). Oxygen dependence of hypericin-induced phototoxicity to EMT6 mouse mammary carcinoma cells. Photochem. Photobiol. 55, 831–837. 10.1111/j.1751-1097.1992.tb08531.x 1409890

[B125] Thong P. S. Kho K. W. Zheng W. Harris M. Soo K. C. Olivo M. (2007). Development of a laser confocal endomicroscope for *in vivo* fluorescence imaging. J. Mech. Med. Biol. 7, 11–18. 10.1142/S0219519407002108

[B126] Thong P. S. Olivo M. Chin W. W. Bhuvaneswari R. Mancer K. Soo K. C. (2009). Clinical application of fluorescence endoscopic imaging using hypericin for the diagnosis of human oral cavity lesions. Br. J. Cancer. 101, 1580–1584. 10.1038/sj.bjc.6605357 19809432 PMC2778520

[B127] Thong P. S. Tandjung S. S. Movania M. M. Chiew W. M. Olivo M. Bhuvaneswari R.. (2012). Toward real-time virtual biopsy of oral lesions using confocal laser endomicroscopy interfaced with embedded computing. J. Biomed. Opt. 17:056009. 10.1117/1.JBO.17.5.056009 22612132

[B128] Thong P. S. Watt F. Ren M. Q. Tan P. H. Soo K. C. Olivo M. (2006). Hypericin-photodynamic therapy (PDT) using an alternative treatment regime suitable for multi-fraction PDT. J. Photochem. Photobiol. B. Biol. 82, 1–8. 10.1016/j.jphotobiol.2005.08.002 16203156

[B129] Tian R. Koyabu N. Morimoto S. Shoyama Y. Ohtani H. Sawada Y. (2005). Functional induction and de-induction of P-glycoprotein by St. John's wort and its ingredients in a human colon adenocarcinoma cell line. Drug Metab. Dispos. 33, 547–554. 10.1124/dmd.104.002485 15640377

[B130] Urbanová M. Košuth J. Čellárová E. (2006). Genetic and biochemical analysis of *Hypericum perforatum* L. plants regenerated after cryopreservation. Plant Cell Rep. 25, 140–147. 10.1007/s00299-005-0050-0 16456647

[B131] Urla C. Armeanu-Ebinger S. Fuchs J. Seitz G. (2015). Successful *in vivo* tumor visualization using fluorescence laparoscopy in a mouse model of disseminated alveolar rhabdomyosarcoma. Surg. Endosc. 29, 1105–1114. 10.1007/s00464-014-3770-9 25149634

[B132] Vandenbogaerde A. L. Cuveele J. F. Proot P. Himpens B. E. Merlevede W. J. de Witte P. A. (1997). Differential cytotoxic effects induced after photosensitization by hypericin. J. Photochem. Photobiol. B. Biol. 38, 136–142. 10.1016/S1011-1344(96)07446-5 9203374

[B133] Vandenbogaerde A. L. Geboes K. R. Cuveele J. F. Agostinis P. M. Merlevede W. J. De Witte P. A. (1996). Antitumour activity of photosensitized hypericin on A431 cell xenografts. Anticancer Res. 16, 1619–1625. 8712679

[B134] Vandepitte J. Van Cleynenbreugel B. Hettinger K. Van Poppel H. de Witte P. A. (2010). Biodistribution of PVP-hypericin and hexaminolevulinate-induced PpIX in normal and orthotopic tumor-bearing rat urinary bladder. Cancer Chemother. Pharmacol. 67, 775–781. 10.1007/s00280-010-1375-0 20532886

[B135] Van de Putte M. Marysael T. Fonge H. Roskams T. Cona M. M. Li J.. (2012). Radiolabeled iodohypericin as tumor necrosis avid tracer: diagnostic and therapeutic potential. Int. J. Cancer. 131, E129–E137. 10.1002/ijc.26492 22038886

[B136] Van de Putte M. Ni Y. De Witte P. A. (2008a). Exploration of the mechanism underlying the tumor necrosis avidity of hypericin. Oncol. Rep. 19, 921–926. 10.3892/or.19.4.921 18357376

[B137] Van de Putte M. Wang H. Chen F. de Witte P. A. Ni Y. (2008b). Hypericin as a marker for determination of tissue viability after intratumoral ethanol injection in a murine liver tumor model. Acad. Radiol. 15, 107–113. 10.1016/j.acra.2007.08.008 18078913

[B138] Van de Putte M. Wang H. Chen F. De Witte P. A. Ni Y. (2008c). Hypericin as a marker for determination of tissue viability after radiofrequency ablation in a murine liver tumor model. Oncol. Rep. 19, 927–932. 10.3892/or.19.4.927 18357377

[B139] Wada A. Sakaeda T. Takara K. Hirai M. Kimura T. Ohmoto N.. (2002). Effects of St John's wort and hypericin on cytotoxicity of anticancer drugs. Drug Metab. Pharmacokinet. 17, 467–474. 10.2133/dmpk.17.467 15618698

[B140] Wölfle U. Seelinger G. Schempp C. M. (2014). Topical application of St. John's wort (Hypericum perforatum). Planta Med. 80, 109–120. 10.1055/s-0033-1351019 24214835

[B141] Xie X. Hudson J. B. Guns E. S. (2001). Tumor-specific and photodependent cytotoxicity of hypericin in the human LNCaP prostate tumor model. Photochem. Photobiol. 74, 221–225. 10.1562/0031-8655(2001)0740221TSAPCO2.0.CO2 11547559

[B142] Zahreddine H. Borden K. L. (2013). Mechanisms and insights into drug resistance in cancer. Front. Pharmacol. 4:28. 10.3389/fphar.2013.00028 23504227 PMC3596793

[B143] Zheng Y. Yin G. Le V. Zhang A. Chen S. Liang X.. (2016). Photodynamic-therapy activates immune response by disrupting immunity homeostasis of tumor cells, which generates vaccine for cancer therapy. Int. J. Biol. Sci. 12, 120–132. 10.7150/ijbs.12852 26722223 PMC4679404

[B144] Zupkó I. Kamuhabwa A. R. D'Hallewin M. A. Baert L. De Witte P. A. (2001). *In vivo* photodynamic activity of hypericin in transitional cell carcinoma bladder tumors. Int. J. Oncol. 18, 1099–1105. 10.3892/ijo.18.5.1099 11295062

